# A Cancer Nanovaccine for Co-Delivery of Peptide Neoantigens and Optimized Combinations of STING and TLR4 Agonists

**DOI:** 10.1021/acsnano.3c04471

**Published:** 2024-02-22

**Authors:** Jessalyn J. Baljon, Alexander J. Kwiatkowski, Hayden M. Pagendarm, Payton T. Stone, Amrendra Kumar, Vijaya Bharti, Jacob A. Schulman, Kyle W. Becker, Eric W. Roth, Plamen P. Christov, Sebastian Joyce, John T. Wilson

**Affiliations:** † Department of Biomedical Engineering, Vanderbilt University, Nashville, Tennessee 37235, United States; ‡ Department of Chemical and Biomolecular Engineering, Vanderbilt University, Nashville, Tennessee 37235, United States; § Northwestern University Atomic and Nanoscale Characterization Experimental (NUANCE) Center, Northwestern University, Evanston, Illinois 60208, United States; ∥ Vanderbilt Institute of Chemical Biology, Vanderbilt University Medical Center, Nashville, Tennessee 37232, United States; ⊥ Department of Pathology, Microbiology, and Immunology, Vanderbilt University Medical Center, Nashville, Tennessee 37232, United States; # Department of Veteran Affairs Tennessee Valley Healthcare System, Nashville, Tennessee 37212, United States; □ Vanderbilt Institute for Infection, Immunology, and Inflammation, Vanderbilt University Medical Center, Nashville, Tennessee 37232, United States; ○ Vanderbilt Center for Immunobiology, Vanderbilt University Medical Center, Nashville, Tennessee 37232, United States; △ Vanderbilt-Ingram Cancer Center, Vanderbilt University Medical Center, Nashville, Tennessee 37232, United States

**Keywords:** cancer vaccine, adjuvant synergy, nanoparticle, immune checkpoint blockade, immunotherapy, endosomal escape

## Abstract

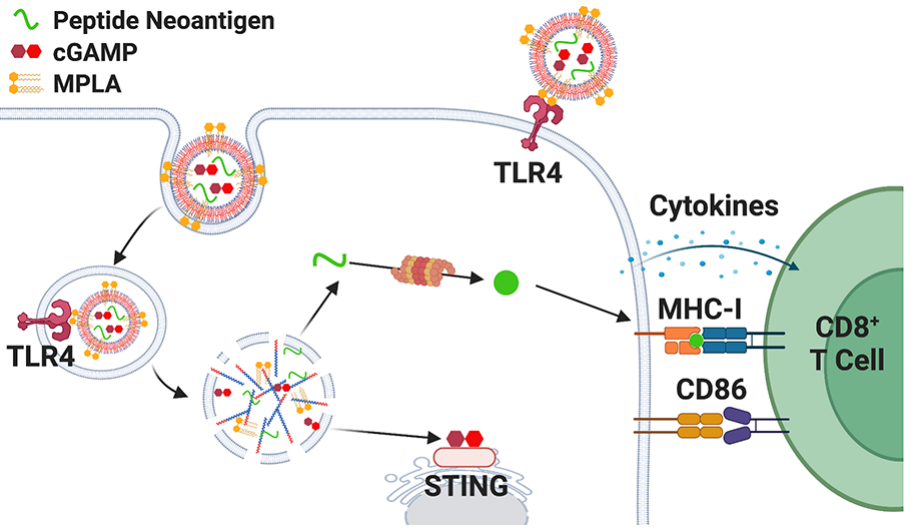

Immune checkpoint blockade (ICB) has revolutionized cancer treatment and led to complete and durable responses, but only for a minority of patients. Resistance to ICB can largely be attributed to insufficient number and/or function of antitumor CD8^+^ T cells in the tumor microenvironment. Neoantigen targeted cancer vaccines can activate and expand the antitumor T cell repertoire, but historically, clinical responses have been poor because immunity against peptide antigens is typically weak, resulting in insufficient activation of CD8^+^ cytotoxic T cells. Herein, we describe a nanoparticle vaccine platform that can overcome these barriers in several ways. First, the vaccine can be reproducibly formulated using a scalable confined impingement jet mixing method to coload a variety of physicochemically diverse peptide antigens and multiple vaccine adjuvants into pH-responsive, vesicular nanoparticles that are monodisperse and less than 100 nm in diameter. Using this approach, we encapsulated synergistically acting adjuvants, cGAMP and monophosphoryl lipid A (MPLA), into the nanocarrier to induce a robust and tailored innate immune response that increased peptide antigen immunogenicity. We found that incorporating both adjuvants into the nanovaccine synergistically enhanced expression of dendritic cell costimulatory markers, pro-inflammatory cytokine secretion, and peptide antigen cross-presentation. Additionally, the nanoparticle delivery increased lymph node accumulation and uptake of peptide antigen by dendritic cells in the draining lymph node. Consequently, nanoparticle codelivery of peptide antigen, cGAMP, and MPLA enhanced the antigen-specific CD8^+^ T cell response and delayed tumor growth in several mouse models. Finally, the nanoparticle platform improved the efficacy of ICB immunotherapy in a murine colon carcinoma model. This work establishes a versatile nanoparticle vaccine platform for codelivery of peptide neoantigens and synergistic adjuvants to enhance responses to cancer vaccines.

Immune checkpoint blockade (ICB) has transformed immunotherapy for a wide variety of cancers, resulting in long-term and durable responses for a subset of patients.^1^ However, many, if not most, patients do not respond to currently approved ICB antibodies for a multitude of reasons, including a low endogenous T cell response to tumor antigens and/or an insufficient number of tumor antigen-specific CD8^+^ T cells that infiltrate into solid tumors.^2−4^ A promising strategy to increase the efficacy of ICB therapy is to employ therapeutic cancer vaccines to prime and/or expand tumor-specific CD8^+^ T cells that can be reactivated in response to ICB.^5−9^ However, cancer vaccines have typically demonstrated a limited capacity to generate antigen-specific T cell responses in patients, historically leading to poor therapeutic outcomes in the clinic.^10−12^ The recent discovery of peptide neoantigens—antigens that arise from mutations in cancer cells—has revitalized interest in therapeutic cancer vaccines. Since neoantigens are only expressed on cancer cells, immunization can lead to a more potent effector T cell response with a significantly reduced risk of autoimmune responses against self-antigen.^13^ As such, neoantigen peptide-based cancer vaccines have been tested in a number of clinical trials.^6,10,12^ In these trials, biopsies of the patient’s tumor and nonmalignant tissue are subjected to whole-exome sequencing to find mutated genes in the tumor. Human leukocyte antigen typing is then required to determine which mutations result in the presentation of potentially immunogenic epitopes. Major histocompatibility complex class I (MHC-I) epitopes are predicted computationally, and these peptides are then synthesized and injected into a patient in a vaccine formulation. In several of the cancer vaccine trials, vaccination induced a detectable antigen-specific T cell response; however, very few have had meaningful clinical benefit.^14^ This could in part be due to the fact that peptide-based vaccines often suffer from weak immunogenicity, leading to poor effector T cell responses. The poor immunogenicity of peptide antigens can be attributed to several intertwined pharmacological barriers, including rapid clearance, poor uptake by dendritic cells (DCs), low accumulation in secondary lymphoid organs (i.e., lymph nodes), inefficient cross-presentation on MHC-I, and potentially, inappropriate choice of immunostimulatory adjuvant(s).^15−18^

Adjuvants are essential to enhancing immune responses to peptide antigens. Adjuvants are included in cancer vaccines to stimulate the innate immune system to induce costimulatory molecule expression on dendritic cells and pro-inflammatory cytokine secretion, which enhance priming and activation of antigen-specific CD8^+^ T cells.^19−21^ While early cancer vaccine clinical trials used adjuvants approved for use in infectious disease vaccines that elicit humoral responses (e.g., MF59), it is now recognized that such adjuvants tend to result in poor cellular immunity, prompting more recent use of agonists of pattern recognition receptors (PRRs) such as CpG DNA (TLR9), monophosphoryl lipid A (MPLA; TLR4), and imiquimod (TLR7).^22−24^ Notably, recent neoantigen clinical trials have used a soluble mixture of a pool of peptide neoantigens and the adjuvant poly-ICLC, a polycation/nucleic acid polyplex that activates TLR3 and MDA-5.^25,26^ Such phase-I trials provide promising proof-of-concept for neoantigen-targeted vaccines. Nonetheless, they yield only suboptimal CD8^+^ T cell responses against a fraction of the antigenic targets with minimal evidence of therapeutic efficacy.^12^ This may, in part, be related to suboptimal vaccine formulation as we, and others, have demonstrated that copackaging of antigen and adjuvant into a common carrier enhances cellular immune responses to vaccines.^27−35^ Collectively, these challenges in antigen and adjuvant delivery motivate the development and optimization of cancer vaccines to augment CD8^+^ T cell responses to peptide neoantigens.

To address this unmet need, we and others have turned to pathogens for inspiration in vaccine design.^36,37^ For example, we recently evaluated codelivery of peptide antigens and the stimulator of interferon genes (STING) agonist cGAMP, an adjuvant that induces a type I interferon (IFN-I) response and has been shown to enhance the antigen-specific CD8^+^ T cell response.^38−41^ We showed that dual-delivery of peptide antigen and cGAMP in endosomolytic polymer vesicles (polymersomes) can induce a robust cytotoxic CD8^+^ T cell response.^33^ The polymersomes were assembled using poly[(ethylene glycol)-*block*-[(2-diethylaminoethyl methacrylate)-*co*-(butyl methacrylate)-*co*-(pyridyl disulfide ethyl methacrylate)]] (PEG-DBP) diblock copolymers. Akin to viruses that escape endo/lysosomal degradation, protonation of diethylaminoethyl methacrylate (DEAEMA) groups in acidic endosomes results in nanoparticle disassembly and exposure of cationic DEAEMA and hydrophobic butyl methacrylate (BMA) residues that act cooperatively to destabilize the endosomal membrane and release associated drug cargo to the cytosol. This approach dramatically enhances the immunostimulatory potency of cGAMP, an anionic molecule that is limited by poor cellular uptake and inefficient cytosolic delivery, while also promoting delivery of peptide antigens to the cytosol to promote cross-presentation on MHC-I molecules, which is critical to activate a CD8^+^ T cell response.^42^

When infected with a pathogen, multiple PRR pathways are typically activated to produce a broader and more robust innate immune response and modulate the downstream adaptive immune response, but most cancer vaccines typically only include a single adjuvant.^43^ Therefore, in recent years several groups have begun to explore the use of multiple, potentially synergistic, adjuvants in a single vaccine platform to enhance and/or tune the innate immune response and further improve antigen immunogenicity and vaccine efficacy.^44−52^ Notably, several studies have shown that using STING agonists in combination with other PRR agonists can further augment the innate immune response with potential to enhance response to vaccines.^44−49,53−59^ For example, previous studies have shown that STING agonists used in combination with TLR4 agonists can enhance the IFN-I response, potentially due to amplification of downstream NF-κB and interferon regulatory factor 3 (IRF3) pathways.^53,54,56^ Atukorale et al. found that coencapsulation of a STING agonist, cdGMP, and MPLA in liposomes enhanced IFN-β production and improved therapeutic efficacy in a B16.F10 melanoma model.^54^ Additionally, Hanson et al. found that when they adjuvanted a liposomal-based vaccine with both cdGMP and MPLA it resulted in an enhanced IgG antibody titer.^53^ Therefore, we hypothesized that coordinated codelivery of a STING (cGAMP) and TLR4 (MPLA) agonist could act synergistically to enhance dendritic cell activation and antigen cross-presentation, resulting in a more robust antitumor T cell response and improved vaccine efficacy.

To explore this, we leveraged endosomolytic polymersomes previously investigated in our group,^60^ which provide an ideal carrier to copackage STING and TLR4 agonists in a defined manner: the vesicular structure allows amphiphilic MPLA to be inserted into the polymeric bilayer at high efficiency for surface display and activation of TLR4, while cGAMP in the aqueous core is released cytosolically to activate STING (Figure 1A). We found that confined impingement jet (CIJ) mixing results in highly uniform polymersomes and efficiently coloads peptide antigen, cGAMP, and MPLA at defined ratios. Co-delivery of the antigen and adjuvants using this endosomolytic nanoparticle platform resulted in an increased IFN-I response, activation of dendritic cells, antigen cross-presentation, and generation of antigen-specific CD8^+^ T cells that increased response to anti-PD-1 ICB.

**Figure 1:**
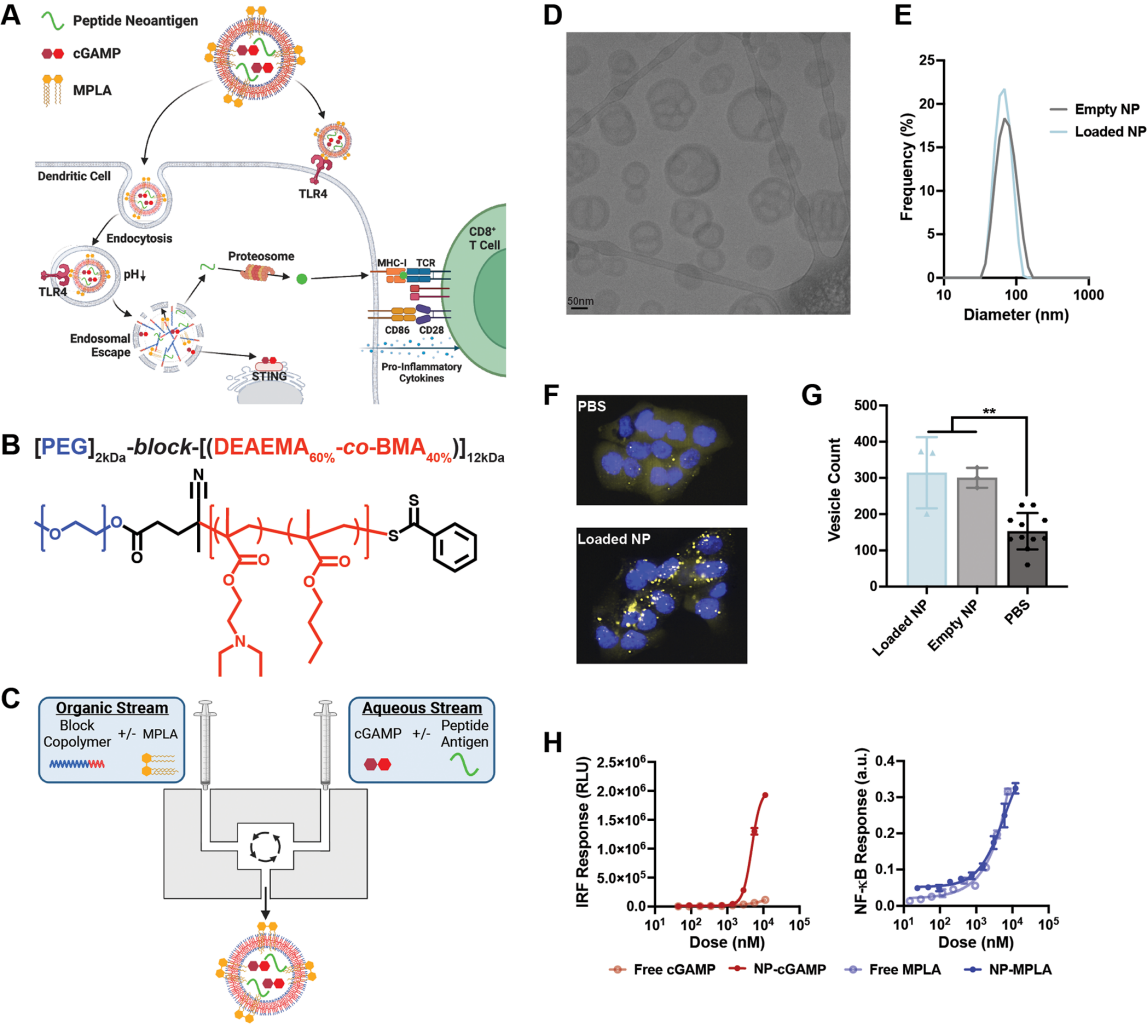
Fabrication and characterization of nanoparticle platform. (A) Schematic representation of coloaded nanoparticle vaccine platform promoting MPLA delivery to cell surface and endosome, and cGAMP and peptide antigen delivery to cytosol via endosomal escape. This enhances innate immune signaling for dendritic cell activation and antigen presentation on MHC-I, which together activate a CD8^+^ T cell response. Made with BioRender.com. (B) Chemical composition and structure of mPEG-*block*-(DEAEMA-*co*-BMA) diblock copolymer. (C) Schematic representation of formulation of nanoparticle via confined impingement jet mixing. Made with BioRender.com. (D) Representative cryogenic electron micrograph of nanoparticle coloaded with antigen, cGAMP, and MPLA. (E) Representative size distribution of empty nanoparticle and nanoparticle coloaded with antigen, cGAMP, and MPLA as measured by dynamic light scattering. (F) Representative fluorescent images of NCI H358 cells expressing a Gal9-mCherry fusion protein after treatment with PBS or the loaded nanoparticle (NP). (G) Vesicle count per cell for NCI H358 cells expressing a Gal9-mCherry fusion protein after treatment with indicated formulations (mean ± SD; *n* = 3–11 biologically independent samples, **P < 0.01; one-way ANOVA with Tukey’s multiple comparisons). (H) *In vitro* evaluation of IRF3 and NF-κB activation after treatment with indicated formulation for 24 h (mean ± SD; *n* = 3 biologically independent samples).

## Results and Discussion

### Formulation of Nanoparticle Vaccine via Confined Impingement Jet Mixing

We first synthesized the pH-responsive diblock copolymer poly[(ethylene glycol)-*block*-[(2-diethylaminoethyl methacrylate)-*co*-(butyl methacrylate)]] (PEG-DB) via reversible addition–fragmentation chain transfer (RAFT) polymerization. The first block is composed of a 2 kDa polyethylene glycol (PEG) chain to impart hydrophilicity and colloidal stability. The second block is a 12 kDa random copolymer composed of DEAEMA and BMA at a 60:40 molar ratio, respectively (Figure 1B, Table S1). At neutral pH, this block is hydrophobic which allows for diblock copolymer self-assembly into vesicular nanoparticles with a hydrophobic bilayer. Additionally, this ratio of DEAEMA to BMA has been previously shown to be optimal for promoting pH-responsive, endosomal escape.^61^ PEG-DB copolymers were self-assembled into vesicular nanoparticles via confined impingement jet (CIJ) mixing (Figure 1C). CIJ mixing utilizes multistream mixers where a diblock copolymer and hydrophobic drug(s) are dissolved in a water miscible organic phase and impinged against an aqueous solution containing hydrophilic drug(s) under turbulent conditions followed by dilution into an aqueous reservoir. The turbulent mixing induces supersaturation and phase separation of copolymer blocks into stable nanoparticles within milliseconds.^62−64^ This technique allows for rapid, scalable formation of monodisperse nanoparticles. While CIJ mixing was done manually with 1 mL syringes throughout our studies, this is a scalable process for nanoparticle fabrication; notably, current COVID-19 mRNA-LNP vaccines from Pfizer are formulated using variations of impingement jet mixing at industrial scales.^65,66^

An important benefit to CIJ mixing is that it allows for simultaneous encapsulation of both hydrophilic and hydrophobic drugs. Since neoantigens are patient-specific, they will vary in charge and hydrophobicity and so it is important that a translatable vaccine platform can efficiently load a large variety of peptides. Peptide antigens, varying in length from 15 to 26 amino acids, were dissolved in the aqueous stream resulting in encapsulation efficiencies ranging from 25 to 70% (Table S2). To further optimize peptide loading efficiency, it is possible that in the future peptide properties can be “normalized” to achieve equal and efficient loading of any desired peptide sequence; for example, by adding a sequence of charged or hydrophobic amino acids proximal to the epitope. cGAMP was added to the aqueous stream and had an encapsulation efficiency of 30–40%, and MPLA was added to the organic stream with an encapsulation efficiency of 90–100%.

Through cryoTEM imaging and dynamic light scattering (DLS), we found that this formulation method formed vesicular nanoparticles that were consistently 70–90 nm in diameter with a low polydispersity index (PDI) of 0.1–0.2 (Figure 1D, E). These are ideal physical properties for vaccine delivery as they enable lymphatic drainage and lymph node accumulation after injection, and uptake by professional antigen presenting cells, such as dendritic cells and macrophages.^67,68^ Importantly, encapsulation of peptides and adjuvants did not affect the nanoparticle size or polydispersity (Figure 1E, Figure S1). This is a major improvement from the first-generation nanovaccine platform from our group, nanoSTING-vax.^33^ This original platform was formulated using a modified direct rehydration method which resulted in nanoparticles ∼200 nm in diameter with a PDI of 0.2–0.3. Additionally, encapsulation of different neoantigen peptides resulted in variation in nanoparticle size and polydispersity.

We also evaluated the ability of the nanoparticle to disrupt the endosomal membrane using a Gal9-mCherry reporter assay.^69^ Gal9-mCherry is a fusion protein of Galectin 9 (Gal9), a protein that binds glycans, and the fluorescent mCherry protein. When the endosome of a cell is disrupted, Gal9-mCherry redistributes from the cytosol to the ruptured endosomes, where it binds the newly exposed glycans. Following treatment with an endosomolytic agent, the number of mCherry puncta can be counted as a measurement of disrupted endosomes and a metric of endosomal escape. We used a Gal9-mCherry expressing H358 non-small cell lung cancer cell line and found that nanoparticle formulation significantly enhanced endosomal disruption compared to PBS (Figure 1F, G). Importantly, loading the nanoparticle with peptide and adjuvants did not affect these endosomal escape capabilities. Overall, we demonstrated here that CIJ mixing can be used to reproducibly encapsulate a variety of antigens and adjuvants in vesicular nanoparticles, and these nanoparticles have physicochemical properties (e.g., < 100 nm diameter, low polydispersity) and endosomal disruption capabilities that are ideal for vaccine delivery.

We then tested the innate immune activity of encapsulated cGAMP (NP-cGAMP) and MPLA (NP-MPLA) (Figure 1H). Since activation of the STING pathway requires delivery of cGAMP to the cytosol, where STING is located, we would expect that nanoparticle delivery of cGAMP would enhance its immunostimulatory activity due to the pH-responsive behavior of the polymer. Consistent with our previous work,^70^ we found that NP-cGAMP significantly enhanced the IFN-I response compared to free cGAMP. TLR4 is localized on the cell surface and/or within endosomes and therefore MPLA does not require cytosolic delivery. Consistent with this notion, we found that loading in the nanoparticle did not affect NP-MPLA’s activity when compared to free MPLA. Overall, the data indicate that pH-responsive polymersomes offer a promising platform to encapsulate cGAMP and MPLA and motivates evaluation of their capacity to synergistically activate innate immunity to enhance peptide immunogenicity *in vivo*.

### Co-Delivery of cGAMP and MPLA Enhances Co-Stimulatory Molecule Expression and Pro-Inflammatory Cytokine Secretion

**Figure 2:**
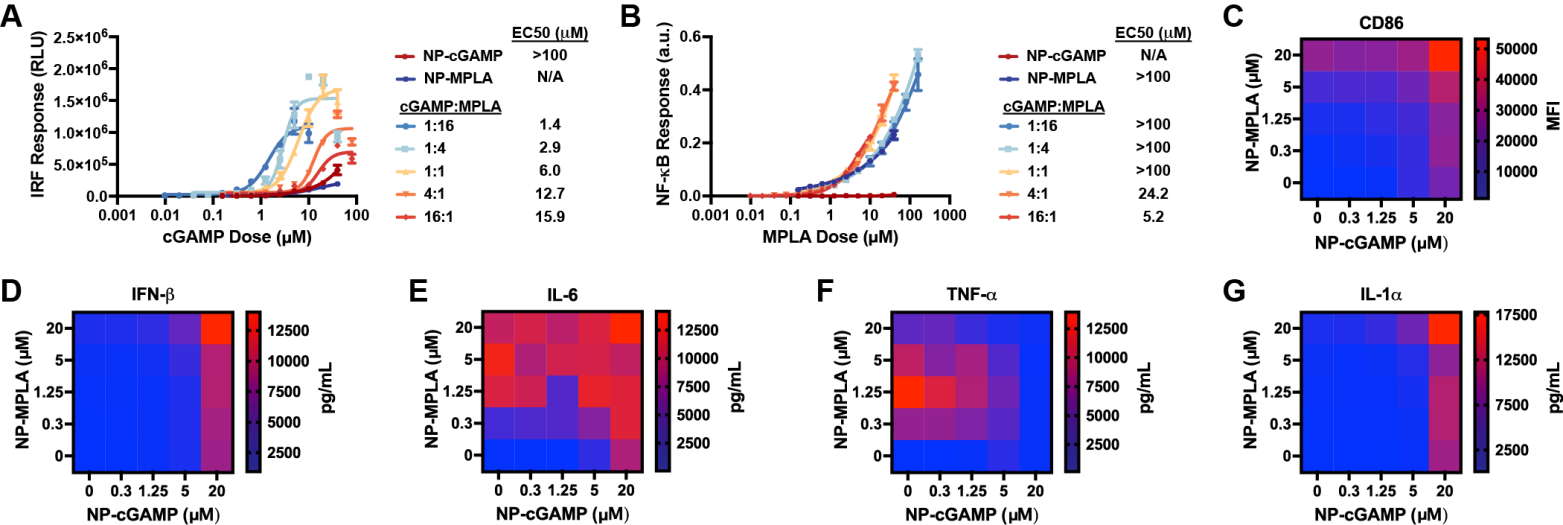
Combining NP-cGAMP and NP-MPLA enhances innate immune activation, co-stimulatory molecule expression, and pro-inflammatory cytokine secretion *in vitro*. (A) *In vitro* evaluation of interferon activation in RAW-Dual reporter cells after treatment with indicated formulation for 24 h, cGAMP doses indicated on *x*-axis, MPLA dose for NP-MPLA dose matched to MPLA dose in cGAMP:MPLA 1:16 (mean ± SD; *n* = 3 biologically independent samples). (B) *In vitro* evaluation of NF-κB activation in RAW-Dual reporter cells after treatment with indicated formulation for 24 h, MPLA doses indicated on *x*-axis, cGAMP dose for NP-cGAMP dose matched to cGAMP dose in cGAMP:MPLA 16:1 (mean ± SD; *n* = 3 biologically independent samples). (C) Flow cytometric quantification of mean fluorescence intensity (MFI) of CD86 expression by BMDCs treated with indicated doses of NP-cGAMP, NP-MPLA, or a mixture of both NPs (*n* = 3 biologically independent samples). (D–G) Concentration of secreted IFN-β (D), IL-6 (E), TNF-α (F), and IL-1α (G) by BMDCs after treatment with indicated doses of NP-cGAMP, NP-MPLA, or a mixture of both NPs (*n* = 3 biologically independent samples).

Since cGAMP and MPLA activate distinct downstream innate immune pathways, we hypothesized that using them in combination could synergistically enhance the dendritic cell activation needed to initiate a robust CD8^+^ T cell response. We first tested nanoparticle encapsulated adjuvants (NP-cGAMP and NP-MPLA) separately to assess synergistic potential prior to coloading them into the same particle. We initially evaluated innate immune responses in RAW-Dual reporter cells, a murine macrophage-like cell line that stably expresses reporter genes for Lucia luciferase and secreted embryonic alkaline phosphatase (SEAP) for quantification of IRF and NF-κB pathway activation, respectively. Comparing NP-cGAMP and NP-MPLA alone to mixtures of NP-cGAMP and NP-MPLA at cGAMP:MPLA molar ratios ranging from 1:16 to 16:1, we showed that addition of NP-MPLA significantly enhanced the IFN-I response over that stimulated by NP-cGAMP alone (Figure 2A), and that the addition of NP-cGAMP slightly enhanced the NF-κB activation over that induced by NP-MPLA alone (Figure 2B). Notably, NP-cGAMP and NP-MPLA alone induced a minimal NF-κB response and IFN-I response, respectively, over the concentration range tested here. These findings further motivated us to leverage adjuvant combinations to shape the innate immune response.

As an important role of adjuvants is to stimulate costimulatory molecule expression and pro-inflammatory cytokine secretion by DCs, we investigated synergy between cGAMP and MPLA in this context. We treated bone marrow-derived dendritic cells (BMDCs) with NP-cGAMP and NP-MPLA at doses ranging from 0 to 20 μM, and mixtures of both nanoparticles at every dose combination within this matrix. We measured the expression of the costimulatory marker CD86 via flow cytometry and found that multiple dose combinations upregulated CD86 expression (Figure 2C, Figure S2A). In previous work using multiple adjuvants there has been limited evaluation of whether two adjuvants are truly synergistic as defined by a mathematical framework, so we applied the Loewe additivity model to determine if the combinations were synergistic, additive, or antagonistic. This model calculates whether the interaction between two different drugs produces a stronger effect than the expected result if both drugs are the same.^71^ A synergy score below −10 is likely to be antagonistic, a score between −10 and 10 is likely to be additive, and a score above 10 is likely to be synergistic. It should be noted that current mathematical models for synergy, including the Loewe model, were originally developed for models of inhibition and cytotoxicity, rather than the activation markers often associated with adjuvant activity; however, using the Loewe model gives an initial indication of the most potent combinations. Calculating the Loewe synergy scores, we found that every combination for CD86 was either additive or synergistic (Figure S3A).

We also measured pro-inflammatory cytokine secretion from BMDCs, specifically screening for IFN-β, IL-6, TNF-α, and IL-1α (Figure 2D–G, Figure S2B–E). We found that secretion of several of these key pro-inflammatory cytokines was enhanced when treated with both adjuvants. The most striking result was with IFN-β, where NP-MPLA produced no effect on its own, but significantly augmented the IFN-β production stimulated by NP-cGAMP. When the Loewe model was applied to these results, most of the cytokines showed additive and synergistic effects (Figure S3B-E). Surprisingly, secretion of TNF-α appeared to be antagonistic at higher concentrations, which we suspect may be related to its cytotoxicity/apoptotic functions at higher doses since even NP-MPLA alone produced a bell-shaped dose–response curve. Overall, these results indicate that cGAMP and MPLA synergistically enhance several innate immune markers that prognosticate a better antigen-specific CD8^+^ T cell response.

**Figure 3:**
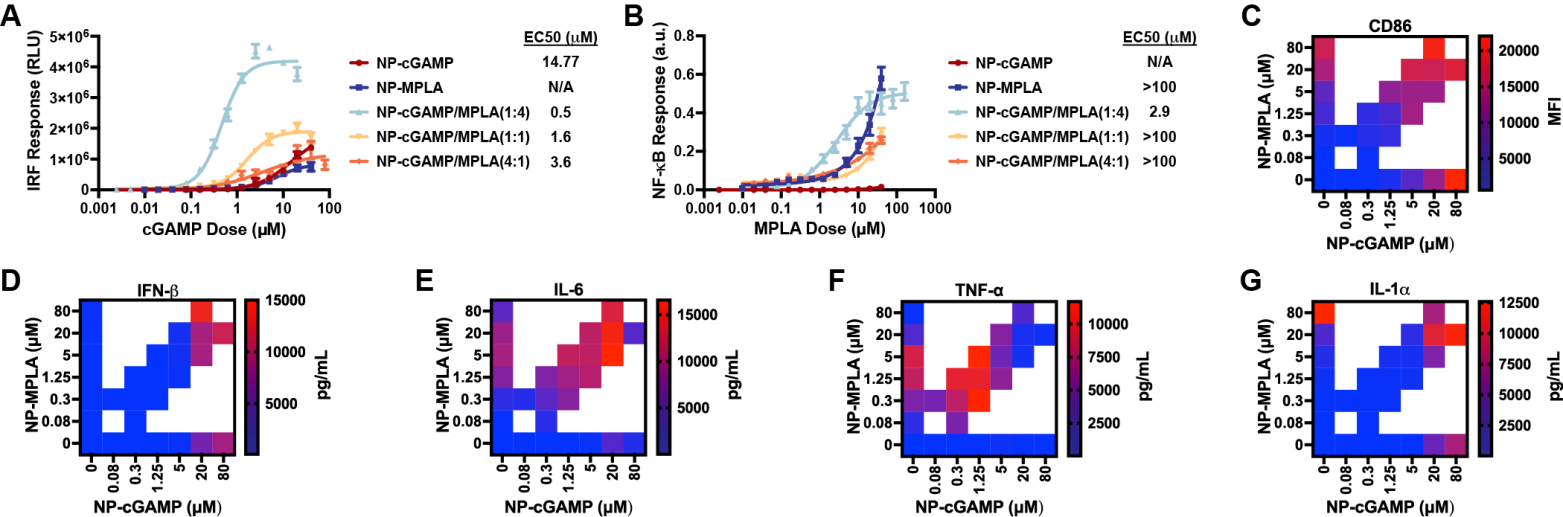
NP-cGAMP/MPLA enhances innate immune activation, co-stimulatory molecule expression, and pro-inflammatory cytokine secretion *in vitro*. (A) *In vitro* evaluation of interferon activation in RAW-Dual reporter cells after treatment with indicated formulation for 24 h, cGAMP doses indicated on *x*-axis, MPLA dose for NP-MPLA dose matched to MPLA dose in NP-cGAMP/MPLA(1:4) (mean ± SD; *n* = 3 biologically independent samples). (B) *In vitro* evaluation of NF-κB activation in RAW-Dual reporter cells after treatment with indicated formulation for 24 h, MPLA doses indicated on *x*-axis, cGAMP dose for NP-cGAMP dose matched to cGAMP dose in NP-cGAMP/MPLA(4:1) (mean ± SD; *n* = 3 biologically independent samples). (C) Flow cytometric quantification of mean fluorescence intensity (MFI) of CD86 expression by BMDCs treated with indicated doses of NP-cGAMP, NP-MPLA, or NP-cGAMP/MPLA (*n* = 3 biologically independent samples). (D–G) Concentration of secreted (D) IFN-β, (E) IL-6, (F) TNF-α, and (G) IL-1α by BMDCs after treatment with indicated doses of NP-cGAMP, NP-MPLA, or NP-cGAMP/MPLA (*n* = 3 biologically independent samples).

Having found that cGAMP and MPLA synergistically enhance BMDC activation when dosed with separate nanoparticles, we wanted to ensure cGAMP and MPLA could be coloaded into the same nanoparticle and that these synergistic effects were maintained. Previous work using multiple adjuvants has rarely focused on the effect of adjuvant ratio, which has been demonstrated to be an important parameter for synergistic delivery of other classes of drugs (e.g., chemotherapeutics). Based on data with separately loaded adjuvants, we chose to move forward with 3 cGAMP:MPLA molar ratios: 1:4, 1:1, and 4:1. We chose these ratios as there was evidence of synergy at these ratios for expression of CD86 and secretion of key pro-inflammatory cytokines. We were able to coload these adjuvants at the selected three ratios into the nanoparticle platform (NP-cGAMP/MPLA) without affecting particle size, polydispersity, and adjuvant encapsulation efficiency (Figure S4). Next, we found that NP-cGAMP/MPLA significantly enhanced activation of the IFN-I and NF-κB pathways in RAW Dual cells, as compared to NP-cGAMP and NP-MPLA, with the 1:4 ratio having the strongest effect, followed by the 1:1 ratio (Figure 3A, B).

Expression of CD86 on BMDCs was also enhanced when treated with NP-cGAMP/MPLA compared to dose-matched NP-cGAMP and NP-MPLA (Figure 3C, S5A). Calculated Loewe synergy scores indicated that the expression of CD86 was a mix of additive and synergistic with many doses at the 1:4 ratio being synergistic (Figure S6A). This indicates that the synergistic effects seen with separately loaded adjuvants were maintained, and in some cases enhanced, with NP-cGAMP/MPLA. It should be noted that treatment with coloaded NP-cGAMP/MPLA significantly lowers the dose of polymer required when compared to mixtures of NP-cGAMP and NP-MPLA, thereby decreasing the potential for adverse or confounding effects related to polymer-mediated toxicity.

Secretion of the same pro-inflammatory cytokines was also measured, which were significantly enhanced when BMDCs were treated with NP-cGAMP/MPLA (Figure 3D–G, S5B–E). Loewe scores showed that NP-cGAMP/MPLA synergistically or additively increased secretion of all cytokines, in a manner that was not strongly ratio dependent (Figure S6B–E). Interestingly, TNF-α was synergistic at doses below 5 μM, most notably at the 1:4 ratio, but was then antagonistic at higher doses for reasons discussed above. Overall, these data indicate that nanoparticles coloaded with cGAMP and MPLA synergistically enhance STING and TLR4 signaling. These findings prompted us to harness the synergistic properties of coloaded nanoparticles to enhance the immunogenicity of peptide antigens, which by themselves are poorly, if at all, immunogenic.

### Nanoparticle Delivery Enhances Lymphatic Accumulation and Uptake by Antigen Presenting Cells

**Figure 4:**
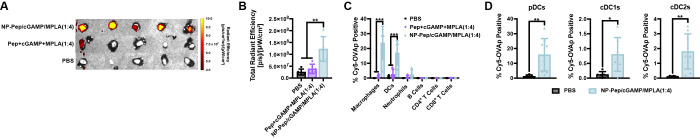
Nanoparticle vaccine enhances lymphatic accumulation and uptake by antigen presenting cells. (A) Fluorescent images of vaccine site draining lymph node 6h after subcutaneous injection of vaccine formulated with Cy5-OVAp. (B) Quantification of fluorescence in vaccine site draining lymph node (mean ± SD; *n* = 5 mice/group; **P < 0.01; one-way ANOVA with Tukey’s multiple comparisons). (C) Percentage of Cy5-OVAp positive cells among immune cell populations in vaccine draining lymph node 6h after injection of vaccine formulated with Cy5-OVAp (mean ± SD; *n* = 5 mice/group; ***P < 0.001; one-way ANOVA with Tukey’s multiple comparisons). (D) Percentage of Cy5-OVAp positive cells among pDCs and cDCs (cDC1 and cDC2) in the vaccine site draining lymph node 6h after injection of vaccine formulated with Cy5-OVAp (mean ± SD; *n* = 6–8 mice/group; *P < 0.05, **P < 0.01; unpaired *t* test).

As cGAMP and MPLA synergistically enhance the expression of CD86 and the secretion of several pro-inflammatory cytokines including IFN-β and IL-6, we developed a complete nanovaccine formulation that coloaded peptide antigens with cGAMP and MPLA: NP-Pep/cGAMP/MPLA. We were able to coload a synthetic long peptide (SLP) derived from the model antigen ovalbumin (OVA) that contains the immunodominant MHC class I SIINFEKL epitope (OVAp), cGAMP, and MPLA into the nanoparticle at the same 3 cGAMP:MPLA molar ratios −1:4, 1:1, 4:1 (Figure S7). This also highlights an important advantage of the CIJ formulation approach and the biphasic structure of the polymersome which together enable coloading of chemically distinct adjuvants (e.g., cGAMP and MPLA) at precisely defined ratios controlled by inlet feed concentrations. Another attractive feature of using a nanoparticle vaccine platform is that nanoparticles smaller than 100 nm in diameter are known to preferentially drain to lymph nodes, where DC and primary T cell activation occurs.^68^ To evaluate this, we formulated NP-Pep/cGAMP/MPLA(1:4) using Cy5-labeled OVAp (Cy5-OVAp) to track it is distribution to the lymph node and uptake by immune cells, compared to a soluble mixture of Cy5-OVAp, cGAMP, and MPLA (Pep+cGAMP+MPLA(1:4)). We subcutaneously vaccinated mice, harvested the draining inguinal lymph node 6h later, and used *exvivo* fluorescence imaging to assess the accumulation of peptide in the lymph node. We found that the nanoparticle vaccine significantly enhanced accumulation of the peptide compared to PBS and the soluble mixture (Figure 4A, B). Importantly, no Cy5-OVAp was detected in the spleen or a nondraining lymph node (Figure S8), suggesting that NPs do not enter the circulation to a detectable level. Additionally, we quantified the uptake of the Cy5-OVAp in immune cell populations—macrophages, DCs, neutrophils, B cells, CD4^+^ T cells, and CD8^+^ T cells—and found that the nanoparticle platform significantly enhanced antigen uptake by macrophages and DCs when compared to the soluble mixture (Figure 4C). We then evaluated uptake of the nanoparticle in DC subsets—plasmacytoid DCs (pDCs, BST^+^CD8^–^) and conventional DCs, cDC1s (CD8^+^) and cDC2s (BST^–^CD8^–^)—and found that all subsets significantly took up antigen compared to the PBS control, with ∼15% of pDCs and 1–2% of cDCs internalizing the antigen (Figure 4D). Although cDC1s are thought of as the most potent cross-presenting DCs, pDCs are known to be a strong producer of IFN-I, which in turn increases overall antigen cross-presentation and CD8^+^ T cell priming.^72,73^ Overall, this finding indicates that NP-Pep/cGAMP/MPLA can enhance antigen accumulation in the lymph node and uptake by antigen presenting cells, which is needed for priming and activation of antigen-specific CD8^+^ T cells.

### Nanoparticle Co-Delivery of Antigen, cGAMP, and MPLA Enhances Antigen-Specific CD8^+^ T Cell Response

**Figure 5:**
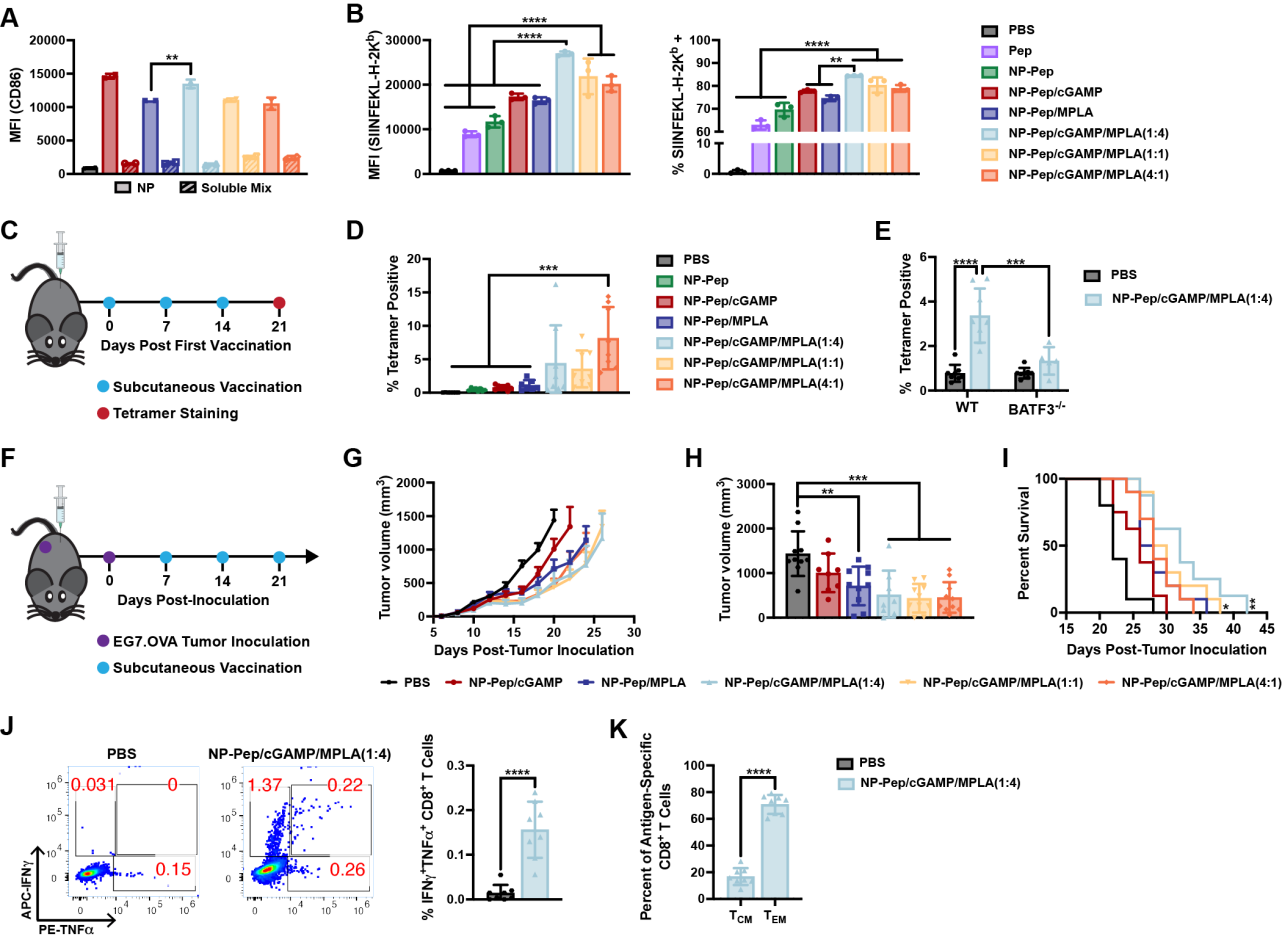
Nanoparticle vaccine enhances dendritic cell activation and cross-presentation, activates antigen-specific CD8^+^ T cells, and provides therapeutic efficacy in the EG7.OVA model. (A) Flow cytometric quantification of mean fluorescence intensity (MFI) of CD86 expression by BMDCs treated with indicated formulations (mean ± SD; *n* = 2; **P < 0.01; one-way ANOVA with Tukey’s multiple comparisons). (B) Flow cytometric quantification of mean fluorescence intensity (MFI) or percentage of SIINFEKL-H2K^b^ positive BMDCs treated with indicated formulations and stained with PE-labeled antibody against SIINFEKL/H-2K^b^ (mean ± SD; *n* = 3; ****P < 0.0001; one-way ANOVA with Tukey’s multiple comparisons). (C) Vaccination and downstream analysis scheme. (D) Quantification of the frequency of SIINFEKL-specific CD8^+^ T cells in spleen after vaccination using peptide/MHC tetramer staining (mean ± SD; *n* = 10 mice/group; ****P < 0.0001; one-way ANOVA with Tukey’s multiple comparisons). (E) Quantification of the frequency of SIINFEKL-specific CD8^+^ T cells in spleen after vaccination using peptide/MHC tetramer staining comparing wild-type mice to Batf3^–/–^ mice (mean ± SD; *n* = 5–8 mice/group; ***P < 0.001, ****P < 0.0001; two-way ANOVA with Tukey’s multiple comparisons). (F) Tumor challenge and therapeutic vaccination scheme. (G) Average EG7.OVA tumor growth in response to indicated formulation (mean ± SEM; *n* = 8–10 mice/group). (H) Average tumor volume on day 20 after tumor inoculation (mean ± SEM; *n* = 8–10 mice/group; **P < 0.01, ***P < 0.001; one-way ANOVA with Tukey’s multiple comparisons). (I) Kaplan–Meier survival curve using 2000 mm^3^ tumor volume as the end point (*n* = 8–10 mice/group; statistical significance between indicated treatment versus PBS and NP-Pep/cGAMP; *P < 0.05, **P < 0.01; Mantel–Cox log-rank test). (J) Representative flow cytometry plots and quantification of IFNγ^+^TNFα^+^ CD8^+^ T cells after *ex vivo* restimulation of splenocytes with SIINFEKL peptide (mean ± SD; *n* = 8 mice/group; ****P < 0.0001; unpaired *t* test). (K) Percentage of central and effector memory antigen-specific CD8^+^ T cells (T_CM_ and T_EM_) in spleen after vaccination (mean ± SD; *n* = 8 mice/group; ****P < 0.0001; unpaired *t* test).

Next, we evaluated whether the complete vaccine formulation (NP-Pep/cGAMP/MPLA) activates dendritic cells by measuring the expression of CD86 on BMDCs after treatment. We compared NP-Pep/cGAMP/MPLA at each cGAMP:MPLA molar ratio (1:4, 1:1, 4:1) to NP-Pep/cGAMP and NP-Pep/MPLA. We found that NP-Pep/cGAMP/MPLA(1:4) significantly enhanced expression of CD86 when compared to NP-Pep/MPLA (Figure 5A). For all the treatment groups the dose of MPLA was maintained, and the dose of cGAMP was adjusted depending on the cGAMP:MPLA ratio. The cGAMP dose for NP-Pep/cGAMP was dose-matched to NP-Pep/cGAMP/MPLA(4:1) and was 16-fold higher than the cGAMP dose for NP-Pep/cGAMP/MPLA(1:4). So, it is unsurprising that NP-Pep/cGAMP/MPLA(1:4) did not significantly enhance expression compared to NP-Pep/cGAMP, and it is notable that it even induced similar expression levels. Additionally, we confirmed that formulations with MPLA, compared to NP-Pep/cGAMP, do not significantly enhance uptake by DCs which could occur via binding of MPLA in the bilayer to TLR4 on the cell surface (Figure S9).

We next tested whether NP-Pep/cGAMP/MPLA enhances antigen cross-presentation on BMDCs. Briefly, we treated BMDCs with free peptide, encapsulated peptide without adjuvants (NP-Pep), NP-Pep/cGAMP, NP-Pep/MPLA, and NP-Pep/cGAMP/MPLA at each cGAMP:MPLA ratio. The presentation of the MHC-I restricted OVA epitope, SIINFEKL, on MHC-I was measured by flow cytometry by using a fluorescently labeled antibody against SIINFEKL bound to H-2K^b^ (Figure 5B). We found that all NP-Pep/cGAMP/MPLA formulations enhanced cross-presentation as compared to PBS, free Pep, and NP-Pep. The 1:4 ratio of cGAMP:MPLA enhanced cross-presentation the most and was significantly higher than NP-Pep/cGAMP and NP-Pep/MPLA. It is unsurprising that free Pep does induce a measurable amount of antigen cross-presentation, as the SLP used, OVAp, can still be degraded by intra- and extracellular proteases and presented on MHC-I, but less efficiently.^42^ Collectively, these data indicate that codelivery of the synergistic adjuvants, cGAMP and MPLA, with peptide neoantigens using a pH-responsive nanoparticle platform can enhance dendritic cell activation and peptide antigen cross-presentation on MHC-I, with the potential to enhance CD8^+^ T cell responses.

We next evaluated the antigen-specific CD8^+^ T cell response *in vivo*. Mice were subcutaneously vaccinated with PBS (vehicle), NP-Pep, NP-Pep/cGAMP, NP-Pep/MPLA, or NP-Pep/cGAMP/MPLA at each ratio on day 0, and then boosted on days 7 and 14 (Figure 5C). On day 21 splenocytes were harvested and the SIINFEKL-specific CD8^+^ T cell response was quantified by pOVA/H-2K^b^ tetramer staining (Figure 5D). We found that NP-Pep generated a minimal (∼0.5%) SIINFEKL-specific CD8^+^ T cell response, with the addition of cGAMP (NP-Pep/cGAMP) and MPLA (NP-Pep/MPLA) generating a stronger response (∼1%). However, copackaging of antigen with both adjuvants (NP-Pep/cGAMP/MPLA) tended to enhance the antigen-specific T cell response (∼5%) at all 3 adjuvant ratios when compared to any of the other formulations.

To evaluate whether cross-presenting DCs were responsible for generating this antigen-specific CD8^+^ T cell response, we repeated this study using Batf3^–/–^ mice, which lack the cross-presenting CD8α^+^ DCs.^74^ We found that the percentage of SIINFEKL-specific CD8^+^ T cells was significantly reduced in Batf3^–/–^ mice after vaccination with NP-Pep/cGAMP/MPLA(1:4) compared to wild type mice (Figure 5E). Collectively, these data demonstrate that incorporating both cGAMP and MPLA into the nanovaccine enhances dendritic cell activation and antigen cross-presentation to promote cellular immunity in response to vaccination with peptide antigens.

### Nanoparticle Vaccine Inhibits Tumor Growth and Improves Survival in Mice

We then evaluated the ability of the platform to enhance therapeutic cancer vaccine efficacy *in vivo* using the EG7.OVA model–a murine thymoma EL-4 cell line that expresses the model OVA antigen. Mice were inoculated subcutaneously in the flank with 3 × 10^5^ EG7.OVA cells on day 0, and then vaccinated on days 7, 14, and 21 with PBS, NP-Pep/cGAMP, NP-Pep/MPLA, or NP-Pep/cGAMP/MPLA at each ratio (Figure 5F). Tumor growth and survival were monitored throughout the study (Figure 5G–I, Figure S10). All NP-Pep/cGAMP/MPLA formulations delayed tumor growth to a significantly greater degree than PBS. Additionally, both NP-Pep/cGAMP/MPLA(1:4) and NP-Pep/cGAMP/MPLA(1:1) significantly prolonged survival as compared to mice that received NP-Pep/cGAMP and PBS. Overall, these data indicate that the nanoparticle vaccine formulation containing both MPLA and cGAMP as adjuvants can improve therapeutic efficacy in a murine tumor model.

### Nanoparticle Vaccine Enhances Functionality of Antigen-Specific CD8^+^ T Cell Response

We next evaluated the functionality and phenotype of the antigen-specific T cell response generated by the vaccine platform. Moving forward, we chose to use only NP-Pep/cGAMP/MPLA(1:4), as this adjuvant ratio was found to be superior or nearly equivalent to the 4:1 and 1:1 ratios in previous studies (e.g., tetramer response, survival benefit), while using a smaller total dose of adjuvant. Mice were subcutaneously vaccinated with PBS (vehicle) or NP-Pep/cGAMP/MPLA(1:4) on days 0, 7, and 14 (Figure 5C). On day 21 the splenocytes were harvested for intracellular cytokine staining and memory phenotyping. Vaccination resulted in a significant increase in the percentage of polyfunctional INFγ^+^TNFα^+^ CD8^+^ T cells in response to restimulation of splenocytes with SIINFEKL (Figure 5J). Next, the memory phenotype of the CD8^+^ T cells in the spleen was evaluated by determining the frequency of central memory T cells (T_CM_, CD44^+^CD62L^+^), a memory CD8^+^ T cell subset that tends to traffic to secondary lymph node organs and persist after an immune response, and effector memory T cells (T_EM_, CD44^+^CD62L^–^), the memory CD8^+^ T cell subset that resides in peripheral circulation and tissues and is more cytolytic and able to immediately recognize and kill target cells.^75^ We found that the nanovaccine generated a significantly stronger antigen-specific T_EM_ response relative to T_CM_, with T_EM_ comprising ∼80% of the tetramer-positive CD8^+^ T cell population (Figure 5K). Overall, this indicates that the vaccine platforms can generate a polyfunctional, antigen-specific T_EM_ response, which is important for killing of cancer cells.

### Nanoparticle Vaccine Enhances Neoantigen Vaccine Efficacy and Response to ICB

**Figure 6:**
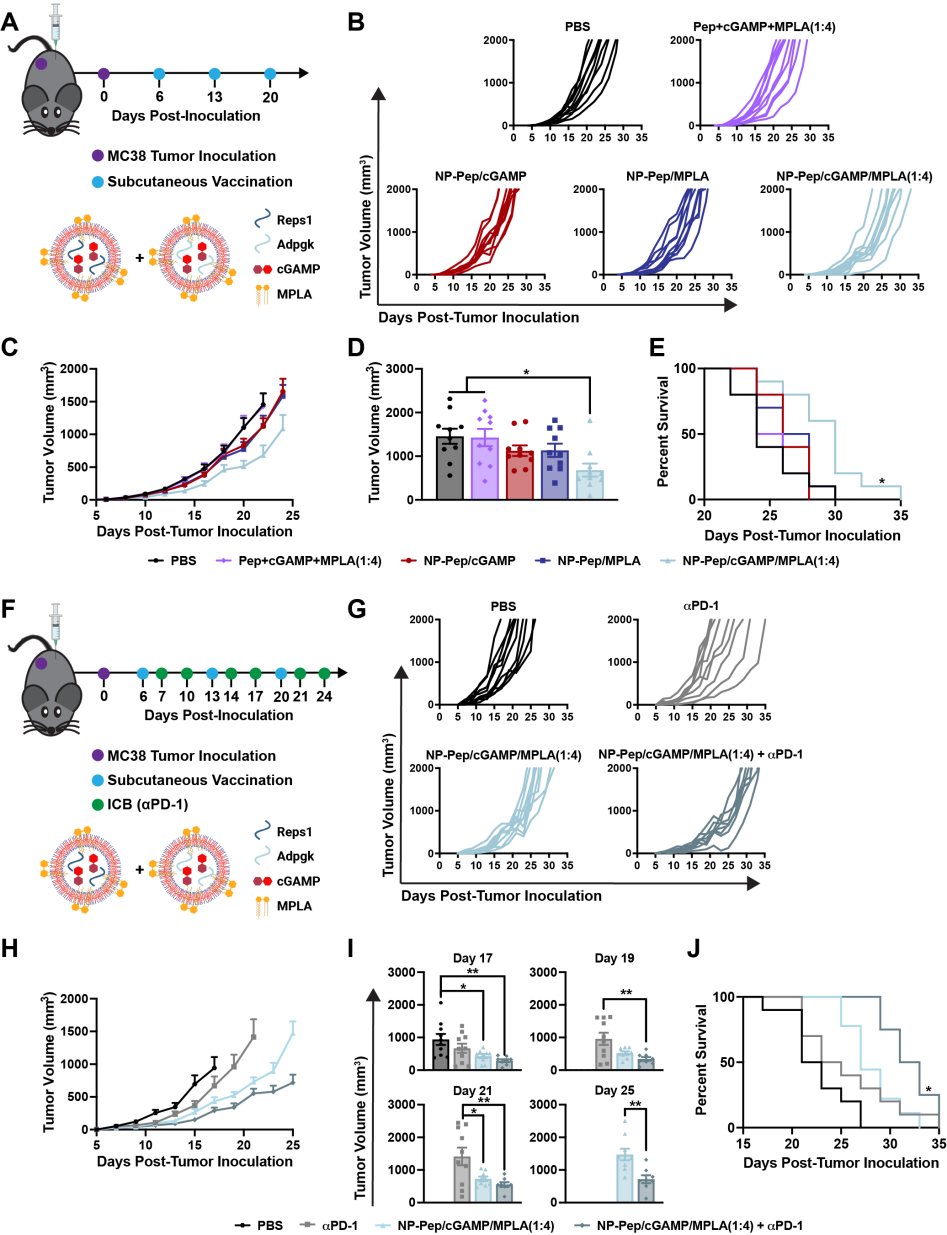
Nanoparticle vaccine provides therapeutic efficacy in MC38 adenocarcinoma model and improves the efficacy of αPD-1 ICB therapy. (A) Tumor challenge and therapeutic vaccination scheme. (B) Spider plots of individual tumor growth curves. (C) Average MC38 tumor growth in response to indicated formulation (mean ± SEM; *n* = 10 mice/group). (D) Average tumor volume on day 22 after tumor inoculation (mean ± SEM; *n* = 10 mice/group; *P < 0.05; one-way ANOVA with Tukey’s multiple comparisons). (E) Kaplan–Meier survival curve using 2000 mm^3^ tumor volume as the end point (*n* = 10 mice/group; statistical significance between NP-Pep/cGAMP/MPLA(1:4) and all other groups shown; *P < 0.05; Mantel–Cox log-rank test). (F) Tumor challenge, therapeutic vaccination, and αPD-1 treatment scheme. (G) Spider plots of individual tumor growth curves. (H) Average MC38 tumor growth in response to indicated formulation (mean ± SEM; *n* = 8–10 mice/group). (I) Average tumor volume on days 17–25 after tumor inoculation (mean ± SEM; *n* = 8–10 mice/group; *P < 0.05, **P < 0.01; one-way ANOVA with Tukey’s multiple comparisons). (J) Kaplan–Meier survival curve using 2000 mm^3^ tumor volume as the end point (*n* = 8–10 mice/group; statistical significance between NP-Pep/cGAMP/MPLA(1:4) + αPD-1 and all other groups shown; *P < 0.05; Mantel–Cox log-rank test).

Based on the data using OVA as a model tumor antigen, we next evaluated the efficacy of the vaccine platform using peptide neoantigens generated from mutations in the MC38 colon cancer cell line. We loaded known and established MC38 neoantigens—Reps1 and Adpgk—into the nanoparticle along with cGAMP and MPLA at a 1:4 ratio. All treatments were a mixture of nanoparticles coloaded with Reps1 and adjuvant(s) and nanoparticles coloaded with Adpgk and adjuvant(s). Mice were inoculated with 1 × 10^6^ MC38 cells, and vaccinated 6 days later, with boosts on days 13 and 20 (Figure 6A). Compared to a soluble mixture of peptides and adjuvants (Pep+cGAMP+MPLA(1:4)) and singly adjuvanted nanoparticle vaccines, we found that only NP-Pep/cGAMP/MPLA(1:4) significantly delayed tumor growth (Figure 6B–D) and improved survival (Figure 6E).

Since NP-Pep/cGAMP/MPLA(1:4) enhanced the antigen-specific CD8^+^ T cell response, we next tested if it could enhance the therapeutic efficacy of ICB. MC38 is known to express PD-L1 but is largely resistant to αPD-1/αPD-L1 therapy.^76^ Hence, we chose to test the therapeutic efficacy of NP-Pep/cGAMP/MPLA(1:4) in combination with αPD-1 therapy in this model. Mice were subcutaneously inoculated with MC38, then vaccinated every 7 days starting at day 6. Additionally, αPD-1 was administered intraperitoneally every 3–4 days (Figure 6F). We found that αPD-1 alone provided minimal therapeutic efficacy, but αPD-1 used in combination with NP-Pep/cGAMP/MPLA(1:4) significantly delayed tumor growth and improved survival (Figure 6G–J). Taken together, these data provide evidence that NP-Pep/cGAMP/MPLA(1:4) provides therapeutic efficacy as a monotherapy and also enhances the efficacy of αPD-1 ICB in a murine colon adenocarcinoma model.

### Nanoparticle Vaccine Activates a Functional Antigen-Specific CD8^+^ T Cell Response Against Neoantigens

**Figure 7:**
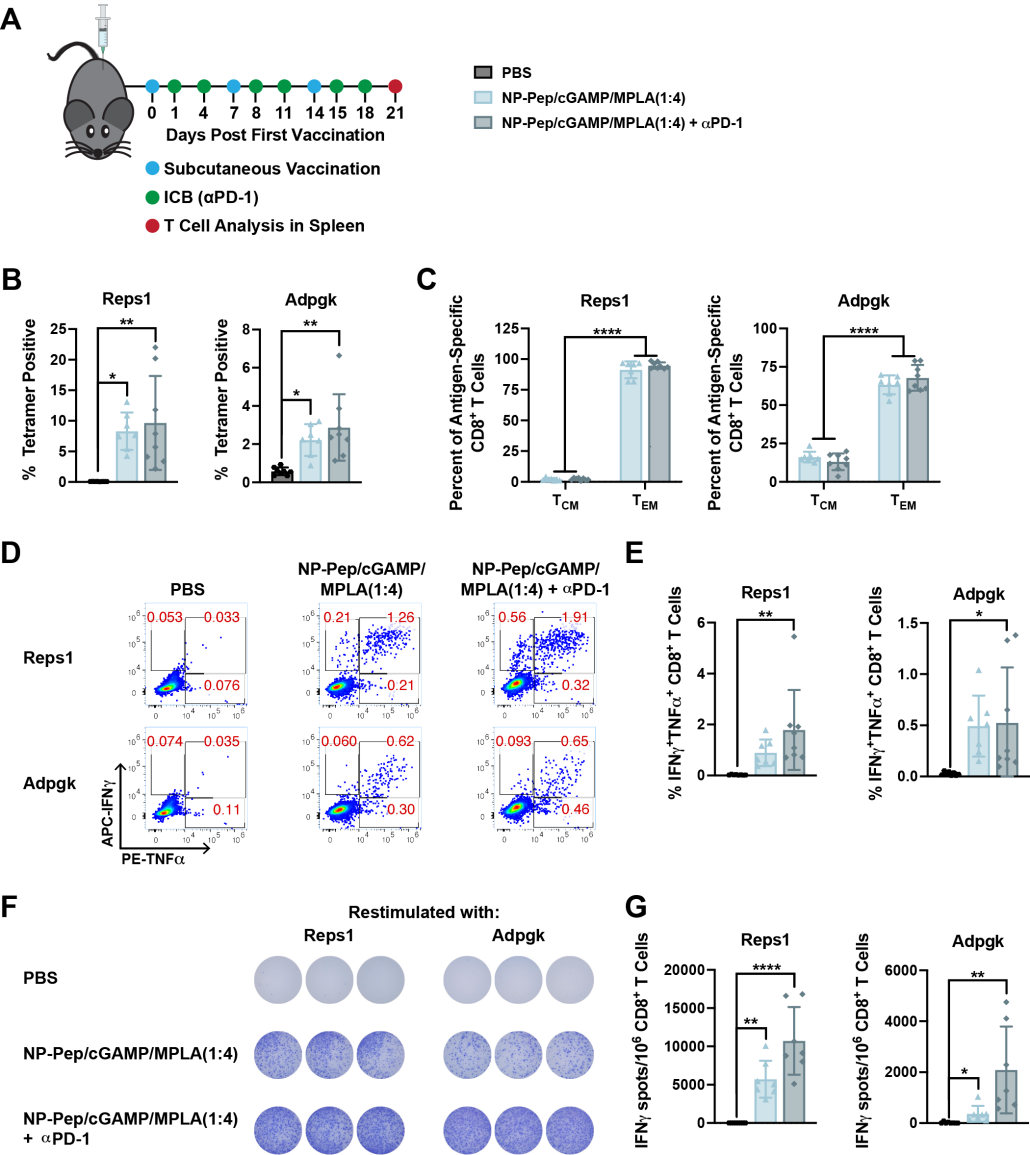
Nanoparticle vaccine activates antigen-specific and functional CD8^+^ T cells against MC38 neoantigens. (A) Vaccination and downstream analysis scheme. (B) Quantification of the frequency of Reps1-specific and Adpgk-specific CD8^+^ T cells in spleen after vaccination using peptide/MHC tetramer staining (mean ± SD; *n* = 7–8 mice/group; *P < 0.05, **P < 0.01; one-way ANOVA with Tukey’s multiple comparisons). (C) Percentage of central and effector memory antigen-specific CD8^+^ T cells in spleen after vaccination (mean ± SD; *n* = 7–8 mice/group; ****P < 0.0001; two-way ANOVA with Tukey’s multiple comparisons). (D) Representative ICCS flow cytometry plots evaluating the percentage of IFNγ^+^TNFα^+^ CD8^+^ T cells after *ex vivo* restimulation of splenocytes with Reps1 (AQLANDVVL) or Adpgk (ASMTNMELM) peptides. (E) Quantification of flow cytometry data in D (mean ± SD; *n* = 7–8 mice/group; *P < 0.05, **P < 0.01; one-way ANOVA with Tukey’s multiple comparisons). (F) Representative ELISPOT wells after *ex vivo* restimulation of splenocytes with Reps1 (AQLANDVVL) or Adpgk (ASMTNMELM) peptides. (G) Quantification of images in F to determine the CD8^+^IFNγ^+^ T cell response (mean ± SD; *n* = 7–8 mice/group; *P < 0.05, **P < 0.01, ****P < 0.0001; one-way ANOVA with Tukey’s multiple comparisons).

After determining that the vaccine platform, in combination with αPD-1 ICB provided therapeutic efficacy, we evaluated the CD8^+^ T cell response and phenotype using the vaccine platform with the MC38 neoantigens. Mice were subcutaneously vaccinated with PBS (vehicle) or NP-Pep/cGAMP/MPLA(1:4) on days 0, 7, and 14, and αPD-1 ICB antibodies were administered very 3–4 days (Figure 7A). On day 21 splenocytes were harvested to evaluate the specificity and functionality of the T cell response. First, the Reps1- and Adpgk-specific CD8^+^ T cell response was evaluated via tetramer staining (Figure 7B). We found that all groups vaccinated with the nanoparticle platform generated a significant antigen-specific CD8^+^ T cell response. We next evaluated the memory phenotype of these antigen-specific CD8^+^ T cells. We found that mice vaccinated with NP-Pep/cGAMP/MPLA(1:4) alone or in combination with αPD-1 induced a stronger Reps1- and Adpgk-specific T_EM_ response than T_CM_ response (Figure 7C). We then investigated the functionality of the T cells via intracellular cytokine staining following antigen restimulation with the Reps1 (AQLANDVVL) or Adpgk (ASMTNMELM) epitope and found that mice treated with NP-Pep/cGAMP/MPLA(1:4) + αPD-1 had significantly enhanced polyfunctional INFγ^+^TNFα^+^ CD8^+^ T cells compared to the PBS control (Figure 7D, E). Finally, we further evaluated the functionality of the T cell response via ELISPOT after restimulation of the splenocytes with either the Reps1 or Adpgk epitope. Again, we found that NP-Pep/cGAMP/MPLA(1:4) significantly enhanced the frequency of IFNγ secreting CD8^+^ T cells (Figure 7F, G). Overall, these data indicate that vaccine platform activates an antigen-specific and functional CD8^+^ T cell response for known murine neoantigens.

## Conclusion

Although ICB has revolutionized cancer immunotherapy, the lack of robust and long-term responses in a subset of patients indicates the need for combination therapy to enhance efficacy. It has been shown that increased antigen-specific CD8^+^ T cell infiltration into tumors is often correlated with improved ICB response rates. This has fueled interest in therapeutic cancer vaccines that can activate an antitumor T cell response, thereby enhancing efficacy of ICB. Here, we describe a nanoparticle vaccine platform rationally designed to enhance the immunogenicity of peptide neoantigens. Nanoparticle cancer vaccines can overcome pharmacological barriers associated with soluble antigen delivery to enhance peptide immunogenicity and improve overall therapeutic efficacy. It is important that these nanoparticle platforms are uniform in size, are amenable to scalable formulation methods, and can incorporate a wide variety of patient-specific neoantigen peptides to improve translatability. We utilized CIJ mixing to formulate pH-responsive vesicular nanoparticles as a versatile nanovaccine platform. Our data show that we can quickly and reproducibly formulate nanoparticles that are monodisperse and less than 100 nm in diameter. Additionally, peptide neoantigens and adjuvants with a range of physical properties can be loaded without impacting nanoparticle properties. Furthermore, by exploring the use of multiple adjuvants, we showed that cGAMP and MPLA can act in synergy to further enhance peptide immunogenicity. Our data demonstrated that cGAMP and MPLA synergistically enhance expression of costimulatory molecules on dendritic cells and secretion of pro-inflammatory cytokines, which are both important for robust priming and activation of T cells. Additionally, we show that the nanoparticle vaccine enhances lymph node accumulation and uptake by antigen presenting cell in the lymph node. Co-loading with peptide neoantigens at 3 defined cGAMP:MPLA ratios—1:4, 1:1, and 4:1—in the nanoparticle platform enhanced cross-presentation of the peptide antigen on MHC-I, which is necessary to induce a CD8^+^ T cell response. In several therapeutic models, the vaccine was able to delay tumor growth and improve survival. Additionally, it improved the therapeutic efficacy of αPD-1 ICB in an MC38 murine tumor model. An important future direction will be to further improve upon the relatively modest therapeutic efficacy of the vaccine; for example, through optimization of dose and regimen, combination with immunomodulators that reprogram the tumor microenvironment, addition of CD4^+^ T cell helper epitopes, and/or incorporation of additional neoantigenic peptides. Taken together, this nanoparticle vaccine is a promising and versatile platform for harnessing adjuvant synergy to enhance peptide antigen immunogenicity, thereby improving the therapeutic efficacy of neoantigen-targeted cancer vaccines.

## Materials and Methods

### Polymer Synthesis and Characterization

4-Cyano-4-(phenylcarbonothioylthio)pentanoic acid (CPADB), N,N′-dicyclohexyl carbodiimide (DCC), 4-(dimethylamino)pyridine (DMAP), butyl methacrylate (BMA), 4,4′-azobis(4-cyanovaleric acid) (V501), poly(ethylene glycol) methyl ether (PEG, MW = 2000 Da), dichloromethane, and 1,4-dioxane were purchased from Sigma-Aldrich. 2-(diethylamino)ethyl methacrylate (DEAEMA) was purchased from TCI chemicals. Methoxyphenol inhibitor was removed from DEAEMA and BMA monomers via gravity filtration through aluminum oxide prior to polymerizations.

Poly[(ethylene glycol)-*block*-[(2-diethylaminoethyl methacrylate)-*co*-(butyl methacrylate)]] was synthesized via RAFT polymerization. A 2 kDa PEG-CPADB macro-RAFT chain transfer agent (mCTA) was synthesized as previously described.^70^ Briefly, PEG (MW = 2000 Da) was coupled to the chain transfer agent CPADB by mixing with DCC and DMAP at a molar ratio of 1:2.5:3:0.15 in dichloromethane and reacting overnight. Unreacted reagent was removed through gravity filtration, and the resulting product was purified by precipitating in cold diethyl ether five times and then vacuum drying. ^1^H NMR was used to calculate the degree of labeling. This mCTA was then dissolved with DEAEMA and BMA monomers (at a 60:40 molar ratio) and the initiator V501 in 1,4-dioxane, purged with nitrogen gas for 30 min, and polymerized for 18h at 70 °C. An mCTA:initiator of 5:1 and a weight fraction of monomer and mCTA to dioxane of 0.4 were used. The resulting polymer was then precipitated five times in cold pentane and vacuum-dried. ^1^H NMR was used to calculate polymer composition, degree of polymerization, and theoretical molecular weight (Figure S11).

### Nanoparticle Formulation

2’3 cGAMP was synthesized as previously described.^70^ MPLA was purchased from Avanti Polar Lipids. Synthetic long peptides (SLPs) containing the epitopes of known neoantigens were purchased from GenScript. The peptides used in this study include the SLP for Ovalbumin, OVAp (SGLEQLESIINFEKL), the MC38 SLP Reps1 (RVLELFRAAQLANDDVVLQIMELC), and the MC38 SLP Adpgk (GIPVHLELASMTNMELMSSIVHQQVF).

Nanoparticles were formulated using CIJ mixing as previously described.^60,62,63^ PEG-DB and MPLA were dissolved in THF and aspirated into a 1 mL polypropylene syringe. Peptide and cGAMP were dissolved in water and aspirated into a separate 1 mL polypropylene syringe. The syringes were then attached to the CIJ unit and impinged against each other by hand into a 20 mL scintillation vial. The total volume impinged was then recollected into both syringes at equal volumes and coimpinged again. This was repeated for a total of five impingements. The final impingement was collected into a 4 mL stirring aqueous reservoir. For all formulations, the concentration of PEG-DB was held constant at 10 mg/mL. For NP-Pep, 500 μg/mL peptide was dissolved in the aqueous phase, for NP-cGAMP 500 μg/mL cGAMP was dissolved in the aqueous phase, and for NP-MPLA 400 μg/mL MPLA was dissolved in the organic phase. For NP-cGAMP/MPLA, cGAMP concentration in the aqueous stream ranged from 200 to 100 μg/mL and MPLA concentration in the organic stream ranged from 100 to 400 μg/mL to ensure the desired cGAMP:MPLA molar ratio was achieved. For all formulations containing peptide antigens, peptide concentration in the aqueous stream was maintained at 500 μg/mL, and cGAMP and MPLA concentrations in their inlet streams were adjusted to achieve the correct ratios.

After the final impingement into the aqueous reservoir, unencapsulated drug was removed via ultracentrifugal filtration through a 50,000 Da MWCO membrane. To quantify peptide and cGAMP loading, an aliquot was removed and diluted in acetonitrile and analyzed via HPLC. OVAp was analyzed using a C18 column (130 Å pore size, 5 μm particle size, 150 × 4 mm inside diameter, ThermoScientific) at 60 °C with a gradient mobile phase from 95% water with 0.1% trifluoroacetic acid (TFA) to 95% acetonitrile with 0.1% TFA over 8 min. Reps1 and Adpgk were analyzed using a Hypersil GOLD C4 column (175 Å pore size, 5 μm particle size, 150 × 4.6 mm inside diameter, ThermoScientific) at 60 °C with a gradient mobile phase from 75% water with 0.1% TFA to 95% acetonitrile with 0.1% TFA over 10 min. cGAMP was analyzed via normal phase HPLC as previously described.^77^ Encapsulation of MPLA was measured using mass spectrometry, with an LTQ XL Linear Ion Trap Mass Spectrometer (ThermoScientific) coupled to an AQUITY Ultra Performance LC (UPLC) (Waters). The mass spectrometer was operated in negative ionization mode. An AQUITY UPLC BEH C8 column (130 Å pore size, 1.7 μm particle size, 150 × 2.1 mm inside diameter, Waters) was used for chromatographic separation at 45 °C. The mobile phase consisted of 2 solvents: solvent A of 10 mM NH_4_OAc in ACN/H_2_O/MeOH 8/1/1 and solvent B of 10 mM NH_4_OAc in MeOH/Iso/ACN 2/2/1. An isocratic flow of 30% solvent A and 70% solvent B was run at 0.3 mL/min for 15 min. The *m*/*z* of 1745.28 was used to quantify MPLA.

Particle diameter and PDI was measured via dynamic light scattering (DLS) by diluting particles in sterile-filtered PBS and characterizing with a Malvern Zetasizer Ultra. For cryoTEM morphology evaluation, 200 mesh Cu grids with a lacey carbon membrane (EMS Cat# LC200-CU-100) were glow-discharged in a Pelco easiGlow glow discharger (Ted Pella Inc., Redding, CA, USA) using an atmosphere plasma generated at 15 mA for 15 s with a pressure of 0.24 mbar. This treatment created a negative charge on the carbon membrane, allowing aqueous samples to spread evenly over of the grid. Four μL of loaded nanoparticles (1 mg/mL stock) was pipetted onto the grid and blotted for 5 s with a blotting pressure of 1, followed by immediate plunging into liquid ethane within an FEI Vitrobot Mark IV plunge freezing instrument (Thermo Fisher Scientific, Waltham, MA, USA). Grids were then transferred to liquid nitrogen for storage. The plunge-frozen grids were kept vitreous at −180 °C in a Gatan Cryo Transfer Holder model 626.5 (Gatan Inc., Pleasanton, CA, USA) while viewing in a JEOL JEM1230 LaB6 emission TEM (JEOL USA, Inc., Peabody, MA,) at 120 keV. Image data was collected by a Gatan Orius SC1000 CCD camera Model 831 (Gatan Inc., Pleasanton, CA, USA).

### Gal9 Recruitment Assay

Gal9 recruitment assays were performed as previously described with minor modifications as follows.^69,78^ NCI-H358 cells stably expressing Gal9-mCherry were seeded in 96-well black walled clear bottom plates (Grenier, catalog number 655090) at a density of 7,500 cells/well and allowed to adhere overnight. The following day, cells were treated with nanoparticle formulations at indicated concentrations and incubated for an additional 24 h. Media was then exchanged with 100 μL of imaging media (FluoroBrite DMEM supplemented with 25 mM HEPES, 10% FBS, 1% Pen/Strep, and 4 μM Hoechst 33342). Cells were imaged using an ImageXpress Nano Automated Imaging System (Molecular Devices) with a 20 x Nikon CFI60 series objective, courtesy of the Vanderbilt High Throughput Screening Core. Images were analyzed using the Transfluor Application Module within the MetaXpress Software (Molecular Devices), which blindly counted the integrated pixel intensity of the Gal9 positive spots within each image.

### *In Vitro* Evaluation of Adjuvant Activity

Activation of IRF and NF-κB pathways were measured in RAW-Dual and THP1-Dual cells (Invivogen). RAW-Dual cells were cultured in DMEM supplemented with 2 mM l-Glutamine, 4.5 g/mL d-glucose, 10% fetal bovine serum (FBS), 100 U/mL penicillin/100 μg/mL streptomycin, and 100 μg/mL normocin. Zeocin (200 μg/mL) was added every other passage to maintain selection pressure. THP1-Dual cells were cultured in RPMI supplemented with 2 mM l-glutamine, 25 mM HEPES, 10% FBS, 100 U/mL penicillin/100 μg/mL streptomycin, and 100 μg/mL normocin. Zeocin (100 μg/mL) and blasticidin (10 μg/mL) were added every other passage to maintain selection pressure. RAW-Dual (50,000 cells/well) or THP1-Dual (20,000 cells/well) were plated in 96-well plates and treated the next day with indicated formulations and concentrations for 24 h. Relative IRF activation was measured using QUANTI-Luc, a lucia luciferase detection reagent, and relative NF-κB activation was measured using QUANTI-blue, a secreted alkaline phosphatase (SEAP) detection reagent, by following manufacturer’s instructions.

### *In Vitro* Evaluation of BMDC Activation

BMDCs were prepared from bone marrow cells harvested from the femurs and tibias from 6 to 8 weeks old C57/Bl6J mice by flushing the bone marrow out with complete BMDC media (RPMI supplemented with 10% FBS, 100 U/mL penicillin/100 μg/mL streptomycin, 2 mM l-glutamine, 10 mM HEPES, 1 mM sodium pyruvate, 1× nonessential amino acids, 55 μM β-mercaptoethanol, 50 μg/mL gentamicin, and 2.5 μg/mL amphotericin B) and passing the cell suspension through a 70 μM cell strainer. Cells were cultured on nontreated 100 × 15 mm Petri dishes in complete BMDC media containing 20 ng/mL granulocyte-macrophage colony-stimulating factor (GM-CSF). Fresh media with 20 ng/mL GM-CSF was added on days 3, 5, and 7. On day 8 differentiation into dendritic cells was confirmed by measuring CD11c expression via flow cytometry using αCD11c (N418, FITC, BioLegend). Cells were then plated on nontreated 12-wells plates in complete BMDC media containing 10 ng/mL GM-CSF at 500,000 cells/well and allowed to adhere overnight. The next day cells were treated with indicated formulations and concentrations for 24 h. Following incubation, the media was removed and stored at −80 °C for cytokine analysis, then cells were scraped into PBS, washed with FACS buffer (PBS supplemented with 1% BSA), incubated with Fc-block (αCD16/32, 2.4G2, Tonbo) for 15 min at 4 °C, then stained with the appropriate antibodies for 1 h at 4 °C. The antibodies used were αCD86 (GL-1, PE/Cy7, BioLegend) and αCD11c (N418, KIRAVIA Blue 520, BioLegend). Cells were then washed with FACS buffer again and then resuspended in DAPI (1 μg/mL in FACS buffer). Flow cytometry was then run on an Amnis CellStream, and median fluorescence intensity (MFI) of CD86 was quantified.

Cytokines secreted into the media was quantified using a LEGENDplex multianalyte flow assay kit (BioLegend) following the manufacturer’s instructions using a v-bottom plate. Data was collected on a Amnis CellStream Flow Cytometer and analyzed using LEGENDplex data analysis software.

### *In Vitro* Evaluation of Antigen Cross-Presentation

BMDCs were harvested and differentiated as described above. Cells were plated on nontreated 12-wells plates in complete BMDC media containing 10 ng/mL GM-CSF at 500,000 cells/well and allowed to adhere overnight. The next day, cells were treated with indicated formulations for 24 h. All treatment groups were 2 μM OVAp, corresponding to 3.75 ng/mL cGAMP in NP-Pep/cGAMP/MPLA(1:4), 15 ng/mL cGAMP in NP-Pep/cGAMP/MPLA(1:1), 60 ng/mL cGAMP in NP-Pep/cGAMP/MPLA(4:1) and NP-Pep/cGAMP, and 45 ng/mL MPLA in all formulations containing MPLA. The next day, cells were scraped, washed with FACS buffer (PBS supplemented with 1% BSA), incubated with Fc-block (αCD16/32, 2.4G2, Tonbo) for 15 min at 4 °C, then stained with αCD11c (N418, APC/Cy7, BioLegend) and αH-2K^b^–SIINFEKL (25-D1.16, PE/Dazzle, BioLegend) for 1 h at 4 °C. Cells were then washed with FACS buffer again, resuspended in DAPI (1 μg/mL in FACS buffer), and analyzed on an Amnis CellStream Flow Cytometer.

### *In Vitro* Evaluation of Peptide Uptake by BMDCs

BMDCs were harvested and differentiated as described above. Cells were plated on nontreated 12-well plates in complete BMDC media containing 10 ng/mL GM-CSF at 500,000 cells/well and allowed to adhere overnight. The next day, cells were treated with indicated formulations for 24 h. All treatment groups were 2 μM Cy5-labeled OVAp, corresponding to 3.75 ng/mL cGAMP in NP-Pep/cGAMP/MPLA(1:4) and NP-Pep/cGAMP, and 45 ng/mL MPLA in NP-Pep/cGAMP/MPLA(1:4). The following day, cells were scraped, washed with FACS buffer (PBS supplemented with 2% FBS), incubated with Fc-block (αCD16/32, 2.4G2, Tonbo) for 15 min at 4 °C, then stained with αCD11c (N418, FITC, BioLegend) for 1 h at 4 °C. Cells were then washed with FACS buffer, resuspended in DAPI (1 μg/mL in FACS buffer), and analyzed on an Amnis CellStream Flow Cytometer.

### Animal Care

Female C57Bl/6J and B6.129S(C)-Batf3^*tm1Kmm*^/J (*Batf3* knockout) mice (6–8 weeks old) were purchased from Jackson Laboratory and housed at Vanderbilt University animal facilities. All animal experiments were reviewed and approved by the Vanderbilt University Institutional Animal Care and Use Committee (IACUC).

### Analysis of Nanoparticle Accumulation and Uptake in Lymph Node

C57/Bl6J mice were injected subcutaneously at the base of the tail with PBS, a soluble mix of Cy5-labeled OVAp, cGAMP, and MPLA, or NP-Pep/cGAMP/MPLA(1:4) loaded with Cy5-labled OVAp. Mice were dosed with 50 μg of OVAp, 0.08 μg of cGAMP, and 0.8 μg of MPLA. 6h after injection, the draining inguinal lymph node, spleen, and axillary lymph node (nondraining) were harvested and Cy5 fluorescence was imaged and measured with the IVIS Lumina III.

Inguinal lymph nodes were mechanically disrupted into single cell suspensions in complete RPMI (cRPMI, RPMI supplemented with 10% FBS, 100 U/mL penicillin/100 μg/mL streptomycin, 2 mM l-glutamine, and 10 mM HEPES) by forcing them through a 70 μm cell strainer. The cells were then stained with Fc-block (αCD16/32, 2.4G2, Tonbo) for 15 min at 4 °C, and then stained with the appropriate antibodies for 30 min at 4 °C. The antibodies used were eFluor 780 viability dye (eBioscience), αTCRβ (H57–597, eFluor 450, eBioscience), αCD4 (RM4–5, SB780, eBioscience), αCD8α (53–6.7, BV605, BioLegend), αCD11b (M1/70, BV510, BioLegend), αCD11c (N418, BV711, BioLegend), αGR-1 (RB6–8C5, PE/Cy7, eBioscience), αF4/80 (BM8, AF488, BioLegend), αCD19 (6D5, PE, BioLegend), and αBST2 (927, PE, BioLegend). Cells were then washed with FACS buffer (PBS supplemented with 2% FBS), fixed with 2% paraformaldehyde for 10 min, washed again with FACS buffer, and then analyzed on a Cytek Aurora flow cytometer (Figure S12, S13).

### Analysis of OVA-Specific CD8^+^ T Cell Response in Spleen

C57/Bl6J mice were injected subcutaneously at the base of the tail on days 0, 7, and 14 with indicated formulations. Mice were dosed with 50 μg of OVAp in all formulations, and 0.8 μg of MPLA in formulations containing MPLA. NP-Pep/cGAMP/MPLA(1:4) mice were dosed with 0.08 μg of cGAMP, NP-Pep/cGAMP/MPLA(1:1) mice were dosed with 0.3125 μg of cGAMP, and NP-Pep/cGAMP/MPLA(4:1) and NP-Pep/cGAMP mice were dosed with 1.25 μg of cGAMP. On day 21, the mice were euthanized to evaluate the CD8^+^ T cell response in the spleen. Spleens were harvested and mechanically disrupted into single cell suspensions in cRPMI by forcing them through a 70 μm cell strainer. Red blood cells were then lysed with ACK lysis buffer, and splenocytes were resuspended in cRPMI and counted.

Splenocytes were plated at 2 × 10^6^ cell/well in a 96-well round-bottom plate, washed twice with FACS buffer (PBS supplemented with 2% FBS and 50 μM dasatinib), stained with Fc-block (αCD16/32, 2.4G2, Tonbo) for 15 min at 4 °C, and then stained with the appropriate antibodies for 1 h at 4 °C. The antibodies used were eFluor 780 viability dye (eBioscience), αCD3e (145–2C11, BV510, BioLegend), and αCD8α (53–6.7, FITC, eBiosceince). The cells were then washed 3 times with FACS buffer and stained for 2 h at 4 °C with 1.5 μg/mL PE-labeled pOVA/H-2K^b^ tetramer. The cells were then washed again with FACS buffer, fixed with 2% paraformaldehyde for 10 min, wash again with FACS buffer, then analyzed on a Cytek Aurora flow cytometer (Figure S14).

### Vaccination and Splenocyte Isolation for Downstream T Cell Analysis Studies

C57/Bl6J mice were injected subcutaneously at the base of the tail on days 0, 7, and 14 with indicated formulations. For studies with OVAp, NP-Pep/cGAMP/MPLA(1:4) contained 50 μg of OVAp, 0.08 μg of cGAMP, and 0.8 μg of MPLA. For studies with MC38 neoantigens, NP-Pep/cGAMP/MPLA(1:4) contained 25 μg of Reps1, 25 μg of Adpgk, 0.08 μg of cGAMP, and 0.8 μg of MPLA. On days 1, 4, 8, 11, 15, and 18, mice were administered 100 μg of αPD-1 (RMP1–14, BioXCell) intraperitoneally for indicated studies. On day 21, the mice were euthanized to evaluate the CD8^+^ T cell response in the spleen. Spleens were harvested and mechanically disrupted into single cell suspensions in cRPMI by forcing them through a 70 μm cell strainer. Red blood cells were then lysed with ACK lysis buffer, and splenocytes were resuspended in cRPMI and counted.

### Establishment of Batf3^–/–^ Model and Evaluation of CD8^+^ T Cell Response

Batf3^–/–^ mice (The Jackson Laboratory) are knockout mice on a C57/Bl6J background that lack exons 1–2 of the *Batf3* gene and are thus deficient in cross-presenting CD8α^+^ conventional dendritic cells (cDC1s).^74^ To validate the model, splenocytes from Batf3^–/–^ and WT mice were harvested as previously described, washed twice with FACS buffer (PBS supplemented with 2% FBS), stained with Fc-block (αCD16/32, 2.4G2, Tonbo) for 15 min at 4 °C, and then stained with the appropriate antibodies for 30 min at 4 °C. The antibodies used were eFluor 780 viability dye (eBioscience), αTCRβ (H57–597, eFluor 450, eBioscience), αCD4 (RM4–5, SB780, eBioscience), αCD8α (53–6.7, BV605, BioLegend), αCD11b (M1/70, BV510, BioLegend), αCD11c (N418, BV711, BioLegend), and αBST2 (927, PE, BioLegend). Cells were then washed with FACS buffer (PBS supplemented with 2% FBS), fixed with 2% paraformaldehyde for 10 min, washed again with FACS buffer, then analyzed on a Cytek Aurora flow cytometer to confirm that Batf3^–/–^ mice maintained the same amount of overall CD8^+^ T cells but lacked cross-presenting cDC1s (Figure S15).

To evaluate the antigen-specific CD8^+^ T cell response, Batf3^–/–^ and WT mice were vaccinated with the OVAp formulation and splenocytes were harvested on day 21 as described above. Splenocytes were incubated with AF647-labeled pOVA/H-2K^b^ tetramer for 1 h at 4 °C followed by two washes with FACS buffer (PBS supplemented with 2% FBS and 50 μM dasatinib). Nonspecific binding was inhibited via incubation in Fc-block (αCD16/32, 2.4G2, Tonbo) for 15 min at 4 °C, followed by staining with the appropriate antibodies for 30 min at 4 °C. The antibodies used were eFluor 780 viability dye (eBioscience), αTCRβ (H57–597, eFluor 450, eBioscience), αCD4 (RM4–5, SB780, eBioscience), and αCD8α (53–6.7, BV605, BioLegend). Cells were then washed twice with FACS buffer, fixed with 2% paraformaldehyde for 10 min, washed again with FACS buffer, then analyzed on a Cytek Aurora flow cytometer (Figure S16).

### Analysis of Memory Phenotype

Mice were vaccinated with either OVAp or MC38 antigens as described above. Splenocytes were incubated with either AF647-labeled pOVA/H-2K^b^ tetramer (NIH Tetramer Core), AF647-labeled Adpgk/H-2D^b^ tetramer (NIH Tetramer Core), or APC-labeled Reps1/H-2D^b^ tetramer (MBL international corporation) for 1 h at 4 °C followed by two washes in FACS buffer (PBS supplemented with 2% FBS and 50 μM dasatinib). Nonspecific binding was inhibited via incubation in Fc-block (αCD16/32, 2.4G2, Tonbo) for 15 min at 4 °C, followed by staining with the appropriate antibodies for 30 min at 4 °C. The antibodies used were eFluor 780 viability dye (eBioscience), αTCR-β (S33–966, eFluor 450, eBioscience), αCD4 (RM4–5, SB780, eBioscience), αCD8α (53–6.7, AF488, Biolegend), αCD44 (IM7, PerCP, BioLegend), and αCD62L (MEL-14, BV711, BioLegend). Cells were then washed twice with FACS buffer, fixed with 2% paraformaldehyde for 10 min, washed again with FACS buffer, then analyzed on a Cytek Aurora flow cytometer (Figure S17).

### Intracellular Cytokine Staining of Splenocytes

Mice were vaccinated with either OVAp or MC38 antigens and splenocytes were harvested on day 21 as described above. Splenocytes were plated at 2 × 10^6^ cell/well in a 96-well round-bottom plate in the presence or absence of 10 mg/mL indicated peptides and incubated for 90 min at 37 °C in a CO_2_ incubator. Cells were then incubated with BD GolgiPlug containing Brefeldin for 5 h at 37 °C in a CO_2_ incubator, then washed, then stained with the appropriate surface antibodies for 45 min at 4 °C. The antibodies used were eFluor 780 viability dye (eBioscience), αCD3e-(145.2C11, PE-Cy7, Biolegend), αCD8α (53–6.7, APC-Cy7, Tonbo), and αCD4 (RM4–5, AF488, Biolegend). The cells were then fixed and permeabilized using the BD Cytofix/Cytoperm fixation/permeabilization kit (BD Biosciences) according to manufacturer’s instructions, and then cells were incubated with αIFNγ (XMG1.2, APC, BD Biosciences) and αTNFα (MP6-XT22, PE, BD Biosciences) antibodies or corresponding isotype controls. Finally, cells were washed and resuspended in FACS buffer, then analyzed on a Cytek Aurora flow cytometer (Figure S18).

### Enzyme-Linked Immunosorbent Spot Assay (ELISpot)

The ELISpot assays were performed using ImmunoSpot mouse IFNg single-color kit from Cellular Technology Limited using the manufacturer’s protocol. Briefly, splenocytes were harvested as above, and 3 × 10^5^ (for Adgpk stimulation) or 1.5 × 10^5^ (for Reps1 stimulation) splenocytes were cultured in IFNγ precoated plates in the presence or absence of corresponding peptides for 48 h at 37 °C in a CO_2_ incubator. Following steps as specified by the manufacturer, plates were washed and incubated in a detection solution containing detection antibodies. After washing the detection solution, the plates were incubated in tertiary solution supplied by the manufacturer, followed by washing and incubation in blue developer solution. Plates were air-dried overnight and scanned using CTL S6 Universal-V Analyzer and ImmunoSpot software. Spots were counted by BioSpot v7.0.26.0 and the counts adjusted to obtain counts/10^6^ cells.

### Tumor Studies with EG7.OVA

For the EG7.OVA therapeutic tumor model, C57Bl/6J mice were inoculated via subcutaneous flank injection with 3 × 10^5^ EG7.OVA cells. Mice were subcutaneously vaccinated at the base of the tail on days 7, 14, and 21 with the following groups: PBS, NP-Pep/cGAMP, NP-Pep/MPLA, NP-Pep/cGAMP/MPLA(1:4), NP-Pep/cGAMP/MPLA(1:1), or NP-Pep/cGAMP/MPLA(4:1). Mice were dosed with 50 μg of OVAp in all formulations, and 0.8 μg of MPLA in formulations containing MPLA. NP-Pep/cGAMP/MPLA(1:4) mice were dosed with 0.08 μg of cGAMP, NP-Pep/cGAMP/MPLA(1:1) mice were dosed with 0.3125 μg cGAMP, and NP-Pep/cGAMP/MPLA(4:1) and NP-Pep/cGAMP mice were dosed with 1.25 μg of cGAMP. Tumor size was measured 3 times a week with calipers using the formula V = (LxWxW)/2. Mice were euthanized at the tumor burden end point of 2000 mm^3^.

### Tumor Studies with MC38

For the MC38 therapeutic model, C57/Bl6J mice were inoculated via subcutaneous flank injection with 1 × 10^6^ MC38 cells. Mice were then vaccinated subcutaneously at the base of tail of days 6, 13, and 20 with the following groups: PBS, soluble mix (Reps1, Adpgk, cGAMP, and MPLA), NP-Pep/cGAMP, NP-Pep/MPLA, and NP-Pep/cGAMP/MPLA(1:4). All groups were dosed with 25 μg of Reps1 and 25 μg of Adpgk, 0.08 μg of cGAMP, and 0.8 μg of MPLA. Tumor volume was monitored as described above and mice were euthanized at the tumor burden end point of 2000 mm^3^.

For the MC38 therapeutic model with ICB, mice were inoculated as described above. On days 6, 13, and 20 mice were vaccinated with either PBS or NP-Pep/cGAMP/MPLA(1:4) with 25 μg of Reps1, 25 μg of Adpgk, 0.08 μg of cGAMP, and 0.8 μg of MPLA. On days 7, 10, 14, 17, 21, and 24, mice were administered 100 μg of αPD-1 (RMP1–14, BioXCell) intraperitoneally. Tumor growth was monitored as described above, and mice were euthanized at the tumor burden end point of 2000 mm^3^.

### Statistical Analysis

Significance for each experiment was calculated as described in the figure caption using GraphPad Prism version 9.3.1. * *P* < 0.05, ** *P* < 0.01, *** *P* < 0.001, **** *P* < 0.001. Error bars represent standard deviation unless otherwise noted. Synergy was calculated using the Loewe method on the SynergyFinder web application.^71^

## Supporting Information Available

Supplementary data describing polymer characterization, nanovaccine drug loading and sizing properties, BMDC costimulatory molecule expression and pro-inflammatory cytokine secretion, Loewe synergy scores, cellular uptake, tumor growth curves, and flow cytometry gating strategies (PDF)

## References

[ref1] Korman A. J.; Garrett-Thomson S. C.; Lonberg N. The Foundations of Immune Checkpoint Blockade and the Ipilimumab Approval Decennial. Nat. Rev. Drug Discov 2022, 21, 509–528. 10.1038/s41573-021-00345-8. 34937915

[ref2] Sharma P.; Allison J. P. The Future of Immune Checkpoint Therapy. Science 2015, 348, 56–61. 10.1126/science.aaa8172. 25838373

[ref3] Shum B.; Larkin J.; Turajlic S. Predictive Biomarkers for Response to Immune Checkpoint Inhibition. Semin Cancer Biol. 2022, 79, 4–17. 10.1016/j.semcancer.2021.03.036. 33819567

[ref4] Jenkins R. W.; Barbie D. A.; Flaherty K. T. Mechanisms of Resistance to Immune Checkpoint Inhibitors. Br. J. Cancer 2018, 118, 9–16. 10.1038/bjc.2017.434. 29319049 PMC5765236

[ref5] Ye Z.; Qian Q.; Jin H.; Qian Q. Cancer Vaccine: Learning Lessons from Immune Checkpoint Inhibitors. J. Cancer 2018, 9, 263–268. 10.7150/jca.20059. 29344272 PMC5771333

[ref6] Lin M. J.; Svensson-Arvelund J.; Lubitz G. S.; Marabelle A.; Melero I.; Brown B. D.; Brody J. D. Cancer Vaccines: The Next Immunotherapy Frontier. Nat. Cancer 2022, 3, 911–926. 10.1038/s43018-022-00418-6. 35999309

[ref7] Curran M. A.; Glisson B. S. New Hope for Therapeutic Cancer Vaccines in the Era of Immune Checkpoint Modulation. Annu. Rev. Med. 2019, 70, 409–424. 10.1146/annurev-med-050217-121900. 30379596

[ref8] Shae D.; Baljon J. J.; Wehbe M.; Becker K. W.; Sheehy T. L.; Wilson J. T. At the Bench: Engineering the Next Generation of Cancer Vaccines. J. Leukoc Biol. 2020, 108, 1435–1453. 10.1002/JLB.5BT0119-016R. 31430398

[ref9] Kleponis J.; Skelton R.; Zheng L. Fueling the Engine and Releasing the Break: Combinational Therapy of Cancer Vaccines and Immune Checkpoint Inhibitors. Cancer Biol. Med. 2015, 12, 201–208. 10.7497/j.issn.2095-3941.2015.0046. 26487965 PMC4607816

[ref10] Shemesh C. S.; Hsu J. C.; Hosseini I.; Shen B. Q.; Rotte A.; Twomey P.; Girish S.; Wu B. Personalized Cancer Vaccines: Clinical Landscape, Challenges, and Opportunities. Mol. Ther 2021, 29, 555–570. 10.1016/j.ymthe.2020.09.038. 33038322 PMC7854282

[ref11] Zhao J.; Chen Y.; Ding Z. Y.; Liu J. Y. Safety and Efficacy of Therapeutic Cancer Vaccines Alone or in Combination with Immune Checkpoint Inhibitors in Cancer Treatment. Front Pharmacol 2019, 10, 1184 10.3389/fphar.2019.01184. 31680963 PMC6798079

[ref12] Blass E.; Ott P. A. Advances in the Development of Personalized Neoantigen-Based Therapeutic Cancer Vaccines. Nat. Rev. Clin Oncol 2021, 18, 215–229. 10.1038/s41571-020-00460-2. 33473220 PMC7816749

[ref13] Schumacher T. N.; Scheper W.; Kvistborg P. Cancer Neoantigens. Annu. Rev. Immunol. 2019, 37, 173–200. 10.1146/annurev-immunol-042617-053402. 30550719

[ref14] Janes M. E.; Gottlieb A. P.; Park K. S.; Zhao Z.; Mitragotri S. Cancer Vaccines in the Clinic. Bioeng Transl Med. 2024, 9, e10588 10.1002/btm2.10588. 38193112 PMC10771564

[ref15] Baljon J. J.; Wilson J. T. Bioinspired Vaccines to Enhance Mhc Class-I Antigen Cross-Presentation. Curr. Opin Immunol 2022, 77, 102215 10.1016/j.coi.2022.102215. 35667222 PMC9695705

[ref16] Liu W.; Tang H.; Li L.; Wang X.; Yu Z.; Li J. Peptide-Based Therapeutic Cancer Vaccine: Current Trends in Clinical Application. Cell Prolif 2021, 54, e13025 10.1111/cpr.13025. 33754407 PMC8088465

[ref17] Stephens A. J.; Burgess-Brown N. A.; Jiang S. Beyond Just Peptide Antigens: The Complex World of Peptide-Based Cancer Vaccines. Front Immunol 2021, 12, 696791 10.3389/fimmu.2021.696791. 34276688 PMC8279810

[ref18] Yewdell J. W. Designing CD8+ T Cell Vaccines: It’s Not Rocket Science (Yet). Curr. Opin Immunol 2010, 22, 402–410. 10.1016/j.coi.2010.04.002. 20447814 PMC2908899

[ref19] Khong H.; Overwijk W. W. Adjuvants for Peptide-Based Cancer Vaccines. J. Immunother Cancer 2016, 4, 56 10.1186/s40425-016-0160-y. 27660710 PMC5028954

[ref20] Gouttefangeas C.; Rammensee H. G. Personalized Cancer Vaccines: Adjuvants Are Important, Too. Cancer Immunol Immunother 2018, 67, 1911–1918. 10.1007/s00262-018-2158-4. 29644387 PMC11028305

[ref21] Temizoz B.; Kuroda E.; Ishii K. J. Vaccine Adjuvants as Potential Cancer Immunotherapeutics. Int. Immunol. 2016, 28, 329–338. 10.1093/intimm/dxw015. 27006304 PMC4922024

[ref22] Kaur A.; Baldwin J.; Brar D.; Salunke D. B.; Petrovsky N. Toll-Like Receptor (TLR) Agonists as a Driving Force Behind Next-Generation Vaccine Adjuvants and Cancer Therapeutics. Curr. Opin Chem. Biol. 2022, 70, 102172 10.1016/j.cbpa.2022.102172. 35785601

[ref23] Hu H. G.; Li Y. M. Emerging Adjuvants for Cancer Immunotherapy. Front Chem. 2020, 8, 601 10.3389/fchem.2020.00601. 32850636 PMC7406886

[ref24] Paston S. J.; Brentville V. A.; Symonds P.; Durrant L. G. Cancer Vaccines, Adjuvants, and Delivery Systems. Front Immunol 2021, 12, 627932 10.3389/fimmu.2021.627932. 33859638 PMC8042385

[ref25] Keskin D. B.; Anandappa A. J.; Sun J.; Tirosh I.; Mathewson N. D.; Li S.; Oliveira G.; Giobbie-Hurder A.; Felt K.; Gjini E.; et al. Neoantigen Vaccine Generates Intratumoral T Cell Responses in Phase Ib Glioblastoma Trial. Nature 2019, 565, 234–239. 10.1038/s41586-018-0792-9. 30568305 PMC6546179

[ref26] Ott P. A.; Hu Z.; Keskin D. B.; Shukla S. A.; Sun J.; Bozym D. J.; Zhang W.; Luoma A.; Giobbie-Hurder A.; Peter L.; Chen C.; Olive O.; Carter T. A.; Li S.; Lieb D. J.; Eisenhaure T.; Gjini E.; Stevens J.; Lane W. J.; Javeri I.; et al. An Immunogenic Personal Neoantigen Vaccine for Patients with Melanoma. Nature 2017, 547, 217–221. 10.1038/nature22991. 28678778 PMC5577644

[ref27] Swartz M. A.; Hirosue S.; Hubbell J. A. Engineering Approaches to Immunotherapy. Science Translational Medicine 2012, 4, 148rv149 10.1126/scitranslmed.3003763. 22914624

[ref28] Knight F. C.; Gilchuk P.; Kumar A.; Becker K. W.; Sevimli S.; Jacobson M. E.; Suryadevara N.; Wang-Bishop L.; Boyd K. L.; Crowe J. E. Jr.; Joyce S.; Wilson J. T. Mucosal Immunization with a pH-Responsive Nanoparticle Vaccine Induces Protective CD8(+) Lung-Resident Memory T Cells. ACS Nano 2019, 13, 10939–10960. 10.1021/acsnano.9b00326. 31553872 PMC6832804

[ref29] Kuai R.; Ochyl L. J.; Bahjat K. S.; Schwendeman A.; Moon J. J. Designer Vaccine Nanodiscs for Personalized Cancer Immunotherapy. Nat. Mater. 2017, 16, 489–496. 10.1038/nmat4822. 28024156 PMC5374005

[ref30] Li A. W.; Sobral M. C.; Badrinath S.; Choi Y.; Graveline A.; Stafford A. G.; Weaver J. C.; Dellacherie M. O.; Shih T. Y.; Ali O. A.; Kim J.; Wucherpfennig K. W.; Mooney D. J. A Facile Approach to Enhance Antigen Response for Personalized Cancer Vaccination. Nat. Mater. 2018, 17, 528–534. 10.1038/s41563-018-0028-2. 29507416 PMC5970019

[ref31] Liu H.; Moynihan K. D.; Zheng Y.; Szeto G. L.; Li A. V.; Huang B.; Van Egeren D. S.; Park C.; Irvine D. J. Structure-Based Programming of Lymph-Node Targeting in Molecular Vaccines. Nature 2014, 507, 519–522. 10.1038/nature12978. 24531764 PMC4069155

[ref32] Lynn G. M.; Sedlik C.; Baharom F.; Zhu Y.; Ramirez-Valdez R. A.; Coble V. L.; Tobin K.; Nichols S. R.; Itzkowitz Y.; Zaidi N.; Gammon J. M.; Blobel N. J.; Denizeau J.; de la Rochere P.; Francica B. J.; Decker B.; Maciejewski M.; Cheung J.; Yamane H.; Smelkinson M. G.; et al. Peptide-TLR-7/8a Conjugate Vaccines Chemically Programmed for Nanoparticle Self-Assembly Enhance CD8 T-Cell Immunity to Tumor Antigens. Nat. Biotechnol. 2020, 38, 320–332. 10.1038/s41587-019-0390-x. 31932728 PMC7065950

[ref33] Shae D.; Baljon J. J.; Wehbe M.; Christov P. P.; Becker K. W.; Kumar A.; Suryadevara N.; Carson C. S.; Palmer C. R.; Knight F. C.; Joyce S.; Wilson J. T. Co-Delivery of Peptide Neoantigens and Stimulator of Interferon Genes Agonists Enhances Response to Cancer Vaccines. ACS Nano 2020, 14, 9904–9916. 10.1021/acsnano.0c02765. 32701257 PMC7775800

[ref34] Tornesello A. L.; Tagliamonte M.; Tornesello M. L.; Buonaguro F. M.; Buonaguro L. Nanoparticles to Improve the Efficacy of Peptide-Based Cancer Vaccines. Cancers (Basel) 2020, 12, 1049 10.3390/cancers12041049. 32340356 PMC7226445

[ref35] Viswanath D. I.; Liu H. C.; Huston D. P.; Chua C. Y. X.; Grattoni A. Emerging Biomaterial-Based Strategies for Personalized Therapeutic in Situ Cancer Vaccines. Biomaterials 2022, 280, 121297 10.1016/j.biomaterials.2021.121297. 34902729 PMC8725170

[ref36] Wu S.; Xia Y.; Hu Y.; Ma G. Bio-Mimic Particles for the Enhanced Vaccinations: Lessons Learnt from the Natural Traits and Pathogenic Invasion. Adv. Drug Deliv Rev. 2021, 176, 113871 10.1016/j.addr.2021.113871. 34311014

[ref37] Rosenthal J. A.; Chen L.; Baker J. L.; Putnam D.; DeLisa M. P. Pathogen-Like Particles: Biomimetic Vaccine Carriers Engineered at the Nanoscale. Curr. Opin Biotechnol 2014, 28, 51–58. 10.1016/j.copbio.2013.11.005. 24832075

[ref38] Gutjahr A.; Papagno L.; Nicoli F.; Kanuma T.; Kuse N.; Cabral-Piccin M. P.; Rochereau N.; Gostick E.; Lioux T.; Perouzel E. The STING Ligand cGamp Potentiates the Efficacy of Vaccine-Induced CD8+ T Cells. JCI Insight 2019, 4 (7), e125107 10.1172/jci.insight.125107. 30944257 PMC6483644

[ref39] He Y.; Hong C.; Fletcher S. J.; Berger A. G.; Sun X.; Yang M.; Huang S.; Belcher A. M.; Irvine D. J.; Li J.; Hammond P. T. Peptide-Based Cancer Vaccine Delivery Via the STINGΔTM-cGamp Complex. Adv. Healthc Mater. 2022, 11, e2200905 10.1002/adhm.202200905. 35670244 PMC11117022

[ref40] Li X. D.; Wu J.; Gao D.; Wang H.; Sun L.; Chen Z. J. Pivotal Roles of cGAS-cGAMP Signaling in Antiviral Defense and Immune Adjuvant Effects. Science 2013, 341, 1390–1394. 10.1126/science.1244040. 23989956 PMC3863637

[ref41] Van Herck S.; Feng B.; Tang L. Delivery of STING Agonists for Adjuvanting Subunit Vaccines. Adv. Drug Deliv Rev. 2021, 179, 114020 10.1016/j.addr.2021.114020. 34756942

[ref42] Embgenbroich M.; Burgdorf S. Current Concepts of Antigen Cross-Presentation. Front Immunol 2018, 9, 1643 10.3389/fimmu.2018.01643. 30061897 PMC6054923

[ref43] Tom J. K.; Albin T. J.; Manna S.; Moser B. A.; Steinhardt R. C.; Esser-Kahn A. P. Applications of Immunomodulatory Immune Synergies to Adjuvant Discovery and Vaccine Development. Trends Biotechnol 2019, 37, 373–388. 10.1016/j.tibtech.2018.10.004. 30470547

[ref44] Collier M. A.; Junkins R. D.; Gallovic M. D.; Johnson B. M.; Johnson M. M.; Macintyre A. N.; Sempowski G. D.; Bachelder E. M.; Ting J. P.; Ainslie K. M. Acetalated Dextran Microparticles for Codelivery of STING and TLR7/8 Agonists. Mol. Pharmaceutics 2018, 15, 4933–4946. 10.1021/acs.molpharmaceut.8b00579. PMC6261357 30281314

[ref45] Barman S.; Borriello F.; Brook B.; Pietrasanta C.; De Leon M.; Sweitzer C.; Menon M.; van Haren S. D.; Soni D.; Saito Y.; Nanishi E.; Yi S.; Bobbala S.; Levy O.; Scott E. A.; Dowling D. J. Shaping Neonatal Immunization by Tuning the Delivery of Synergistic Adjuvants Via Nanocarriers. ACS Chem. Biol. 2022, 17, 2559–2571. 10.1021/acschembio.2c00497. 36028220 PMC9486804

[ref46] Kuai R.; Sun X.; Yuan W.; Ochyl L. J.; Xu Y.; Hassani Najafabadi A.; Scheetz L.; Yu M. Z.; Balwani I.; Schwendeman A.; Moon J. J. Dual TLR Agonist Nanodiscs as a Strong Adjuvant System for Vaccines and Immunotherapy. J. Controlled Release 2018, 282, 131–139. 10.1016/j.jconrel.2018.04.041. PMC6056764 29702142

[ref47] Zhang B. D.; Wu J. J.; Li W. H.; Hu H. G.; Zhao L.; He P. Y.; Zhao Y. F.; Li Y. M. STING and TLR7/8 Agonists-Based Nanovaccines for Synergistic Antitumor Immune Activation. Nano Res. 2022, 15, 6328–6339. 10.1007/s12274-022-4282-x. 35464625 PMC9014842

[ref48] Pradhan P.; Toy R.; Jhita N.; Atalis A.; Pandey B.; Beach A.; Blanchard E. L.; Moore S. G.; Gaul D. A.; Santangelo P. J. TRAF6-IRF5 Kinetics, TRIF, and Biophysical Factors Drive Synergistic Innate Responses to Particle-Mediated MPLA-CpG Co-Presentation. Sci. Adv. 2021, 7, abd4235 10.1126/sciadv.abd4235. PMC7806213 33523878

[ref49] Temizoz B.; Kuroda E.; Ohata K.; Jounai N.; Ozasa K.; Kobiyama K.; Aoshi T.; Ishii K. J. TLR9 and STING Agonists Synergistically Induce Innate and Adaptive Type-II IFN. Eur. J. Immunol. 2015, 45, 1159–1169. 10.1002/eji.201445132. 25529558 PMC4671267

[ref50] Kim J. Y.; Rosenberger M. G.; Chen S.; Ip C. K.; Bahmani A.; Chen Q.; Shen J.; Tang Y.; Wang A.; Kenna E.; Son M.; Tay S.; Ferguson A. L.; Esser-Kahn A. P. Discovery of New States of Immunomodulation for Vaccine Adjuvants Via High Throughput Screening: Expanding Innate Responses to PRRs. ACS Cent Sci. 2023, 9, 427–439. 10.1021/acscentsci.2c01351. 36968540 PMC10037445

[ref51] Nihesh N.; Manna S.; Studnitzer B.; Shen J.; Esser-Kahn A. P. A Synthetic Pathogen Mimetic Molecule Induces a Highly Amplified Synergistic Immune Response Via Activation of Multiple Signaling Pathways. Chem. Sci. 2021, 12, 6646–6651. 10.1039/D1SC00964H. 34040739 PMC8132936

[ref52] Taylor D.; Meyer C. T.; Graves D.; Sen R.; Fu J.; Tran E.; Mirza B.; Rodriguez G.; Lang C.; Feng H.; Quaranta V.; Wilson J. T.; Kim Y. J.; Korrer M. J. MuSyC Dosing of Adjuvanted Cancer Vaccines Optimizes Antitumor Responses. Front Immunol 2022, 13, 936129 10.3389/fimmu.2022.936129. 36059502 PMC9437625

[ref53] Hanson M. C.; Crespo M. P.; Abraham W.; Moynihan K. D.; Szeto G. L.; Chen S. H.; Melo M. B.; Mueller S.; Irvine D. J. Nanoparticulate STING Agonists Are Potent Lymph Node-Targeted Vaccine Adjuvants. J. Clin Invest 2015, 125, 2532–2546. 10.1172/JCI79915. 25938786 PMC4497758

[ref54] Atukorale P. U.; Raghunathan S. P.; Raguveer V.; Moon T. J.; Zheng C.; Bielecki P. A.; Wiese M. L.; Goldberg A. L.; Covarrubias G.; Hoimes C. J.; Karathanasis E. Nanoparticle Encapsulation of Synergistic Immune Agonists Enables Systemic Codelivery to Tumor Sites and IFNβ-Driven Antitumor Immunity. Cancer Res. 2019, 79, 5394–5406. 10.1158/0008-5472.CAN-19-0381. 31431457 PMC6801091

[ref55] Pandey S.; Gruenbaum A.; Kanashova T.; Mertins P.; Cluzel P.; Chevrier N. Pairwise Stimulations of Pathogen-Sensing Pathways Predict Immune Responses to Multi-Adjuvant Combinations. Cell Syst 2020, 11, 495–508. 10.1016/j.cels.2020.10.001. 33113356 PMC7677225

[ref56] Andrade W. A.; Agarwal S.; Mo S.; Shaffer S. A.; Dillard J. P.; Schmidt T.; Hornung V.; Fitzgerald K. A.; Kurt-Jones E. A.; Golenbock D. T. Type I Interferon Induction by Neisseria Gonorrhoeae: Dual Requirement of Cyclic GMP-AMP Synthase and Toll-Like Receptor 4. Cell Rep 2016, 15, 2438–2448. 10.1016/j.celrep.2016.05.030. 27264171 PMC5401638

[ref57] Kocabas B. B.; Almacioglu K.; Bulut E. A.; Gucluler G.; Tincer G.; Bayik D.; Gursel M.; Gursel I. Dual-Adjuvant Effect of pH-Sensitive Liposomes Loaded with STING and TLR9 Agonists Regress Tumor Development by Enhancing Th1 Immune Response. J. Controlled Release 2020, 328, 587–595. 10.1016/j.jconrel.2020.09.040. 32971199

[ref58] Toy R.; Keenum M. C.; Pradhan P.; Phang K.; Chen P.; Chukwu C.; Nguyen L. A. H.; Liu J.; Jain S.; Kozlowski G.; Hosten J.; Suthar M. S.; Roy K. TLR7 and RIG-I Dual-Adjuvant Loaded Nanoparticles Drive Broadened and Synergistic Responses in Dendritic Cells in Vitro and Generate Unique Cellular Immune Responses in Influenza Vaccination. J. Controlled Release 2021, 330, 866–877. 10.1016/j.jconrel.2020.10.060. PMC7906919 33160004

[ref59] Hou Y.; Wang Y.; Tang Y.; Zhou Z.; Tan L.; Gong T.; Zhang L.; Sun X. Co-Delivery of Antigen and Dual Adjuvants by Aluminum Hydroxide Nanoparticles for Enhanced Immune Responses. J. Controlled Release 2020, 326, 120–130. 10.1016/j.jconrel.2020.06.021. 32585230

[ref60] Pagendarm H. M.; Stone P. T.; Kimmel B. R.; Baljon J. J.; Aziz M. H.; Pastora L. E.; Hubert L.; Roth E. W.; Almunif S.; Scott E. A.; Wilson J. T. Engineering Endosomolytic Nanocarriers of Diverse Morphologies Using Confined Impingement Jet Mixing. Nanoscale 2023, 15, 16016–16029. 10.1039/D3NR02874G. 37753868 PMC10568979

[ref61] Manganiello M. J.; Cheng C.; Convertine A. J.; Bryers J. D.; Stayton P. S. Diblock Copolymers with Tunable pH Transitions for Gene Delivery. Biomaterials 2012, 33, 2301–2309. 10.1016/j.biomaterials.2011.11.019. 22169826 PMC3266685

[ref62] Allen S.; Osorio O.; Liu Y. G.; Scott E. Facile Assembly and Loading of Theranostic Polymersomes Via Multi-Impingement Flash Nanoprecipitation. J. Controlled Release 2017, 262, 91–103. 10.1016/j.jconrel.2017.07.026. PMC5603398 28736263

[ref63] Han J.; Zhu Z.; Qian H.; Wohl A. R.; Beaman C. J.; Hoye T. R.; Macosko C. W. A Simple Confined Impingement Jets Mixer for Flash Nanoprecipitation. J. Pharm. Sci. 2012, 101, 4018–4023. 10.1002/jps.23259. 22777753 PMC6382459

[ref64] Johnson B. K.; Prud’homme R. K. Mechanism for Rapid Self-Assembly of Block Copolymer Nanoparticles. Phys. Rev. Lett. 2003, 91, 118302 10.1103/PhysRevLett.91.118302. 14525460

[ref65] Daniel S.; Kis Z.; Kontoravdi C.; Shah N. Quality by Design for Enabling RNA Platform Production Processes. Trends Biotechnol 2022, 40, 1213–1228. 10.1016/j.tibtech.2022.03.012. 35491266

[ref66] Warne N.; Ruesch M.; Siwik P.; Mensah P.; Ludwig J.; Hripcsak M.; Godavarti R.; Prigodich A.; Dolsten M. Delivering 3 Billion Doses of Comirnaty in 2021. Nat. Biotechnol. 2023, 41, 183–188. 10.1038/s41587-022-01643-1. 36732478

[ref67] Chang T. Z.; Stadmiller S. S.; Staskevicius E.; Champion J. A. Effects of Ovalbumin Protein Nanoparticle Vaccine Size and Coating on Dendritic Cell Processing. Biomater Sci. 2017, 5, 223–233. 10.1039/C6BM00500D. 27918020 PMC5285395

[ref68] Thomas S. N.; Schudel A. Overcoming Transport Barriers for Interstitial-, Lymphatic-, and Lymph Node-Targeted Drug Delivery. Curr. Opin Chem. Eng. 2015, 7, 65–74. 10.1016/j.coche.2014.11.003. 25745594 PMC4345645

[ref69] Munson M. J.; O’Driscoll G.; Silva A. M.; Lazaro-Ibanez E.; Gallud A.; Wilson J. T.; Collen A.; Esbjorner E. K.; Sabirsh A. A High-Throughput Galectin-9 Imaging Assay for Quantifying Nanoparticle Uptake, Endosomal Escape and Functional RNA Delivery. Commun. Biol. 2021, 4, 211 10.1038/s42003-021-01728-8. 33594247 PMC7887203

[ref70] Shae D.; Becker K. W.; Christov P.; Yun D. S.; Lytton-Jean A. K. R.; Sevimli S.; Ascano M.; Kelley M.; Johnson D. B.; Balko J. M.; Wilson J. T. Endosomolytic Polymersomes Increase the Activity of Cyclic Dinucleotide STING Agonists to Enhance Cancer Immunotherapy. Nat. Nanotechnol 2019, 14, 269–278. 10.1038/s41565-018-0342-5. 30664751 PMC6402974

[ref71] Zheng S.; Wang W.; Aldahdooh J.; Malyutina A.; Shadbahr T.; Tanoli Z.; Pessia A.; Tang J. Synergyfinder Plus: Toward Better Interpretation and Annotation of Drug Combination Screening Datasets. Genomics Proteomics Bioinformatics 2022, 20, 587–596. 10.1016/j.gpb.2022.01.004. 35085776 PMC9801064

[ref72] Mouries J.; Moron G.; Schlecht G.; Escriou N.; Dadaglio G.; Leclerc C. Plasmacytoid Dendritic Cells Efficiently Cross-Prime Naive T Cells in Vivo after TLR Activation. Blood 2008, 112, 3713–3722. 10.1182/blood-2008-03-146290. 18698004 PMC2572799

[ref73] Villadangos J. A.; Young L. Antigen-Presentation Properties of Plasmacytoid Dendritic Cells. Immunity 2008, 29, 352–361. 10.1016/j.immuni.2008.09.002. 18799143

[ref74] Hildner K.; Edelson B. T.; Purtha W. E.; Diamond M.; Matsushita H.; Kohyama M.; Calderon B.; Schraml B. U.; Unanue E. R.; Diamond M. S.; Schreiber R. D.; Murphy T. L.; Murphy K. M. Batf3 Deficiency Reveals a Critical Role for CD8α+ Dendritic Cells in Cytotoxic T Cell Immunity. Science 2008, 322, 1097–1100. 10.1126/science.1164206. 19008445 PMC2756611

[ref75] Kaech S. M.; Wherry E. J.; Ahmed R. Effector and Memory T-Cell Differentiation: Implications for Vaccine Development. Nat. Rev. Immunol 2002, 2, 251–262. 10.1038/nri778. 12001996

[ref76] Taylor M. A.; Hughes A. M.; Walton J.; Coenen-Stass A. M. L.; Magiera L.; Mooney L.; Bell S.; Staniszewska A. D.; Sandin L. C.; Barry S. T.; et al. Longitudinal Immune Characterization of Syngeneic Tumor Models to Enable Model Selection for Immune Oncology Drug Discovery. J. Immunother Cancer 2019, 7, 328 10.1186/s40425-019-0794-7. 31779705 PMC6883640

[ref77] Wang-Bishop L.; Wehbe M.; Shae D.; James J.; Hacker B. C.; Garland K.; Chistov P. P.; Rafat M.; Balko J. M.; Wilson J. T. Potent Sting Activation Stimulates Immunogenic Cell Death to Enhance Antitumor Immunity in Neuroblastoma. J. Immunother Cancer 2020, 8, e000282 10.1136/jitc-2019-000282. 32169869 PMC7069313

[ref78] Kilchrist K. V.; Dimobi S. C.; Jackson M. A.; Evans B. C.; Werfel T. A.; Dailing E. A.; Bedingfield S. K.; Kelly I. B.; Duvall C. L. Gal8 Visualization of Endosome Disruption Predicts Carrier-Mediated Biologic Drug Intracellular Bioavailability. ACS Nano 2019, 13, 1136–1152. 10.1021/acsnano.8b05482. 30629431 PMC6995262

