# Supplementary material for: A Cancer Nanovaccine for Co-Delivery of Peptide Neoantigens and Optimized Combinations of STING and TLR4 Agonists
Source: ACS Nano. 2024 Feb 22;18(9):6845–62. doi: 10.1021/acsnano.3c04471 (PMC10919087; doi:10.1021/acsnano.3c04471)
Supplement: Supplementary file 1 — nn3c04471_si_001.pdf [file nn3c04471_si_001.pdf]

**Supporting Information**:

# A Cancer Nanovaccine for Co-Delivery of Peptide Neoantigens and Optimized Combinations of STING and TLR4 Agonists

*Jessalyn J. Baljon[a], Alexander J. Kwiatkowski[b], Hayden M. Pagendarm[a], Payton T. Stone[b], Amrendra Kumar[e], Vijaya Bharti[b], Jacob A. Schulman[b], Kyle W. Becker[b], Eric W. Roth[c], Plamen P. Christov[d], Sebastian Joyce[e,f,g,h], John T. Wilson[a,b,d,e,g,h,i,*]*

[a]Department of Biomedical Engineering, Vanderbilt University, Nashville, TN 37235, USA
[b]Department of Chemical and Biomolecular Engineering, Vanderbilt University, Nashville, TN 37235, USA
[c]Northwestern University Atomic and Nanoscale Characterization Experimental (NUANCE) Center, Northwestern University, Evanston, IL 60208, USA
[d]Vanderbilt Institute of Chemical Biology, Vanderbilt University Medical Center, Nashville, TN 37232, USA
[e]Department of Pathology, Microbiology, and Immunology, Vanderbilt University Medical Center, Nashville, TN 37232, USA
[f]Department of Veteran Affairs Tennessee Valley Healthcare System, Nashville, TN 37212, USA
[g]Vanderbilt Institute for Infection, Immunology, and Inflammation, Vanderbilt University Medical Center, Nashville, TN 37232, USA
[h]Vanderbilt Center for Immunobiology, Vanderbilt University Medical Center, Nashville, TN 37232, USA
[i]Vanderbilt-Ingram Cancer Center, Vanderbilt University Medical Center, Nashville, TN 37232, USA

*Corresponding Author: john.t.wilson@vanderbilt.edu

## Supplementary Data:

**Table S1. Summary of Polymer Properties**

| PEG (Da) | DEAEMA-co-BMA (Da) | % DEAEMA | % BMA |
|---|---|---|---|
| 2000 | 11,573 | 62.3% | 37.7% |

**Table S2. Summary of Peptide Properties and Encapsulation Efficiencies**

| Peptide | Sequence | GRAVY | Charge | Encapsulation Efficiency |
|---|---|---|---|---|
| **OVAp** | SGLEQLESIINFEKL | -0.01 | -2 | 68.4% |
| **Reps1** | RVLELFRAAQLANDDVVLQIMELC | 0.63 | -2.1 | 26.2% |
| **Adpgk** | GIPVHLELASMTNMELMSSIVHQQVF | 0.55 | -1.8 | 34.5% |

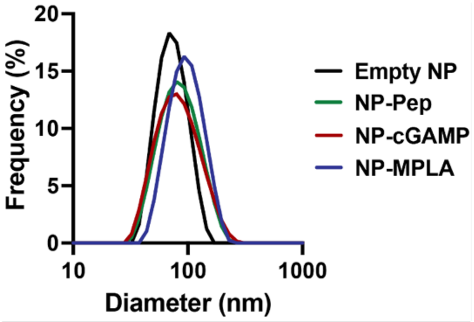


**Figure S1. Characterization of Nanoparticle Size**. Size distribution of empty nanoparticles compared to nanoparticles loaded with peptide antigen, cGAMP, or MPLA, measured by dynamic light scattering.

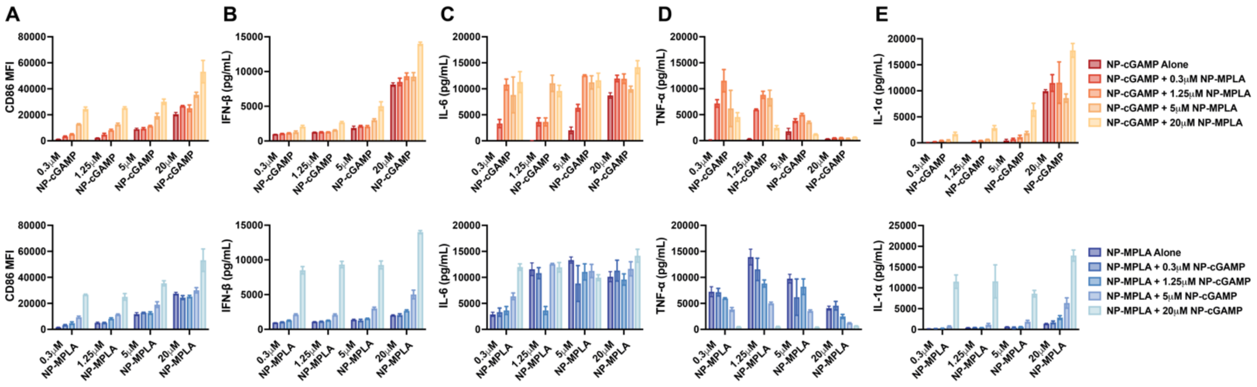


**Figure S2. Dendritic Cell Co-Stimulatory Molecule Expression and Pro-Inflammatory Cytokine Secretion After Treatment with NP-cGAMP + NP-MPLA.** (A) Flow cytometric quantification of mean fluorescence intensity (MFI) of CD86 expression by BMDCs treated with indicated dose of NP-cGAMP (top) or NP-MPLA (bottom) and soluble mixes of both NPs at indicated concentrations (mean +/- SD; n=3 biologically independent samples). (B-E) Concentration of secreted IFN-β (B), IL-6 (C), TNF-α (D), and IL-1α (E) by BMDCs after treatment with indicated dose of NP-cGAMP (top) or NP-MPLA (bottom) and soluble mixes of both NPs at indicated concentrations (mean +/- SD; n=3 biologically independent samples).

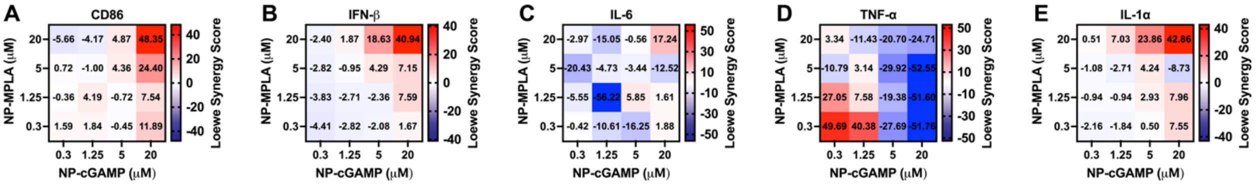


**Figure S3. Loewe Synergy Score for BMDCs Treated with NP-cGAMP + NP-MPLA.** (A) Loewe synergy score of MFI of CD86 expression on BMDCs treated with soluble mixes of NP-cGAMP and NP-MPLA at indicated doses. (B-E) Loewe synergy score of concentration of secreted IFN-β (B), IL-6 (C), TNF-α (D), and IL-1α (E) by BMDCs after treatment with soluble mixes of NP-cGAMP and NP-MPLA at indicated doses.

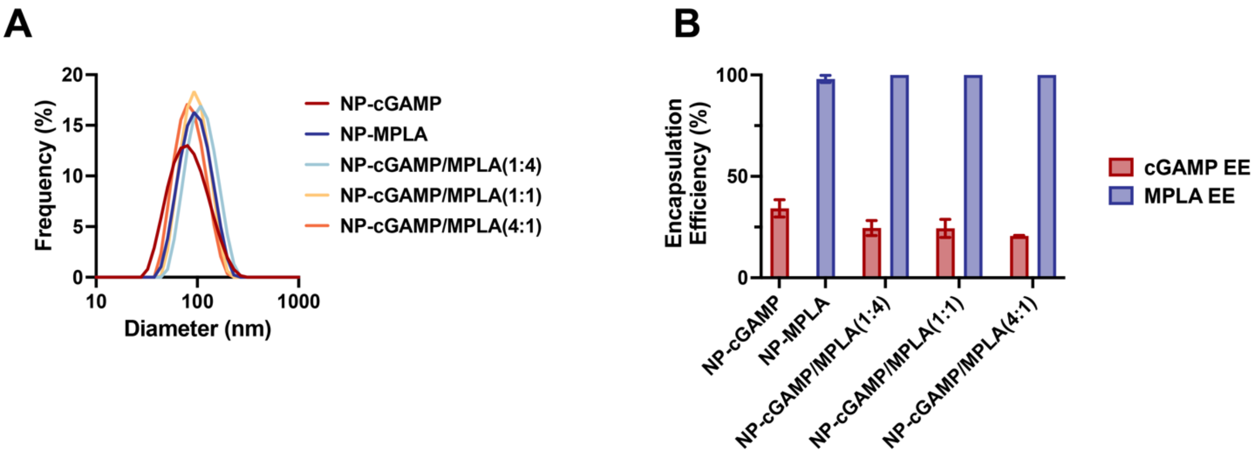


**Figure S4. Characterization of NP-cGAMP/MPLA Size and Encapsulation Efficiency.** (A) Size distribution of indicated formulations measured by dynamic light scattering. (B) Encapsulation efficiency of cGAMP and MPLA in indicated formulations.

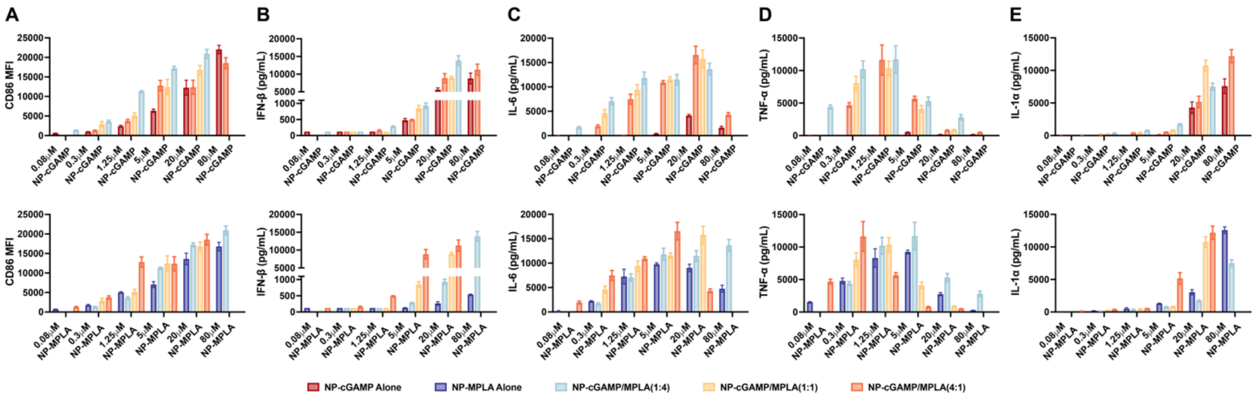


**Figure S5. Dendritic Cell Co-Stimulatory Molecule Expression and Pro-Inflammatory Cytokine Secretion After Treatment with NP-cGAMP/MPLA.** (A) Flow cytometric quantification of mean fluorescence intensity (MFI) of CD86 expression by BMDCs treated with NP-cGAMP, NP-MPLA, or NP-cGAMP/MPLA at 1:4, 1:1, or 4:1 cGAMP:MPLA ratios at indicated dose of cGAMP (top) or MPLA (bottom) (mean +/- SD; n=3 biologically independent samples). (B-E) Concentration of secreted IFN-β (B), IL-6 (C), TNF-α (D), and IL-1α (E) by BMDCs after treatment with NP-cGAMP, NP-MPLA, or NP-cGAMP/MPLA at 1:4, 1:1, or 4:1 cGAMP:MPLA ratios at indicated dose of cGAMP (top) or MPLA (bottom) (mean +/- SD; n=3 biologically independent samples).

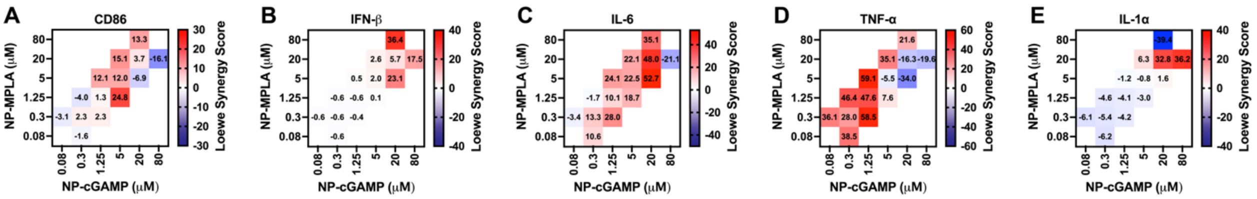


**Figure S6. Loewe Synergy Score for BMDCs Treated with NP-cGAMP/MPLA.** (A) Loewe synergy score of MFI of CD86 expression on BMDCs treated with NP-cGAMP/MPLA at indicated doses of cGAMP and MPLA. (B-E) Loewe synergy score of concentration of secreted IFN-β (B), IL-6 (C), TNF-α (D), and IL-1α (E) by BMDCs after treatment with NP-cGAMP/MPLA at indicated doses of cGAMP and MPLA.

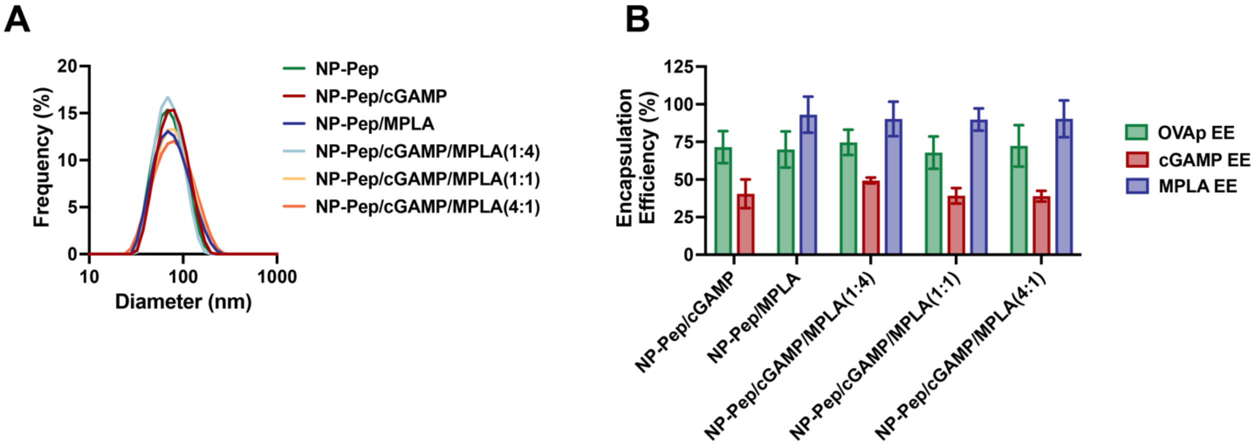


**Figure S7. Characterization of NP-Pep/cGAMP/MPLA Size and Encapsulation Efficiency.** (A) Size distribution of indicated formulations measured by dynamic light scattering. (B) Encapsulation efficiency of OVAp, cGAMP, and MPLA in indicated formulations.

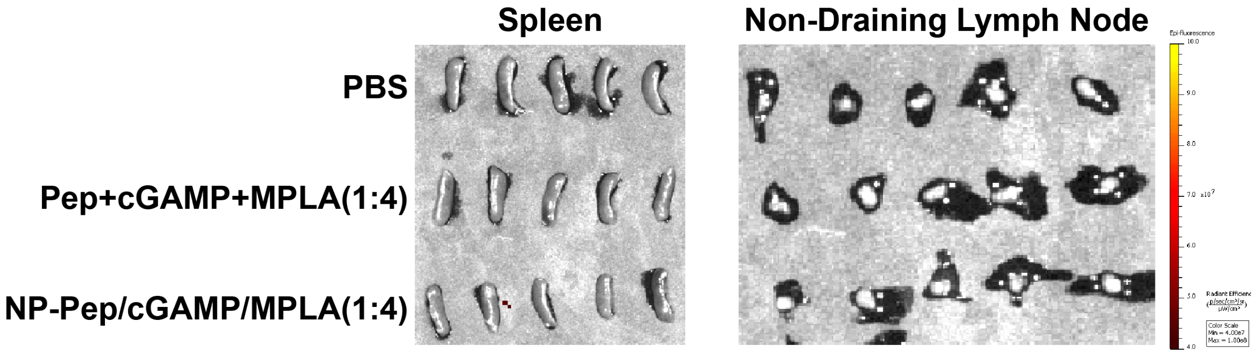


**Figure S8. Accumulation of Antigen Peptide in Spleen and Non-Draining Lymph Node After Vaccination.** Images of spleen and non-draining lymph node 6h after subcutaneous injection of vaccine formulated with Cy5-OVAp.

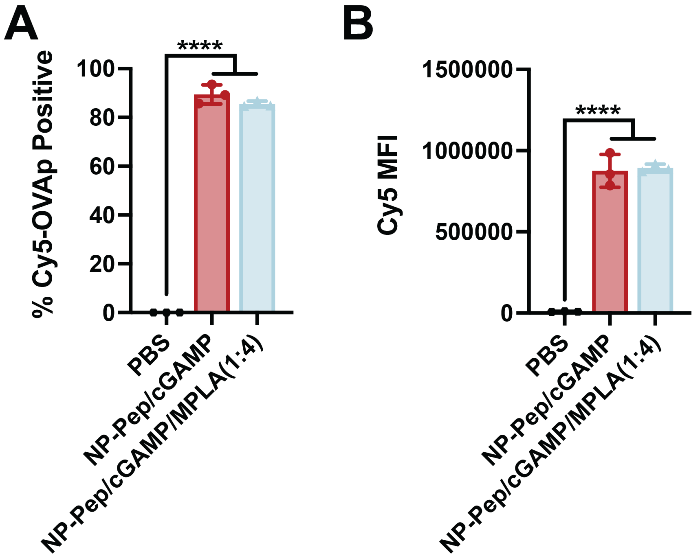


**Figure S9. Peptide Uptake by BMDCs.** Flow cytometric quantification of percentage of Cy5-OVAp positive (A) and mean fluorescence intensity (MFI) of Cy5 signal (B) in BMDCs after treatment with indicated formulations (mean +/- SD; n=3; ****P<0.0001; one-way ANOVA with Tukey's multiple comparisons).

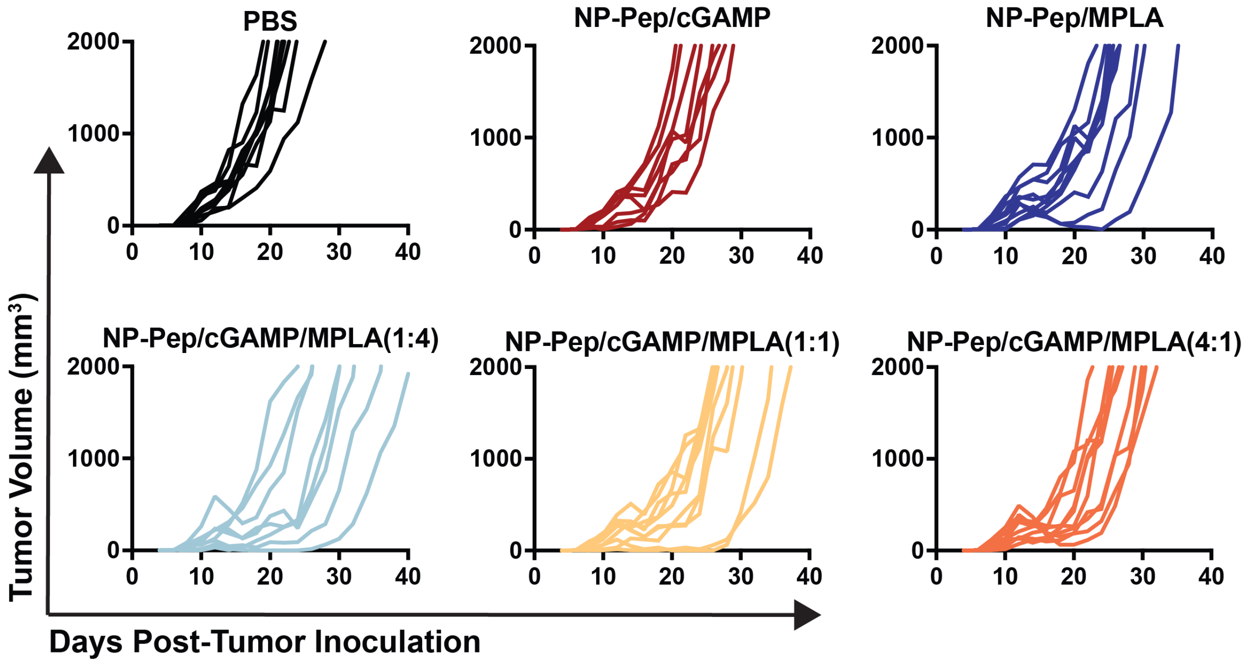


**Figure S10. Tumor Growth Spider Plots.** Spider plots of individual EG7.OVA tumor growth curves in response to treatment with indicated formulation.

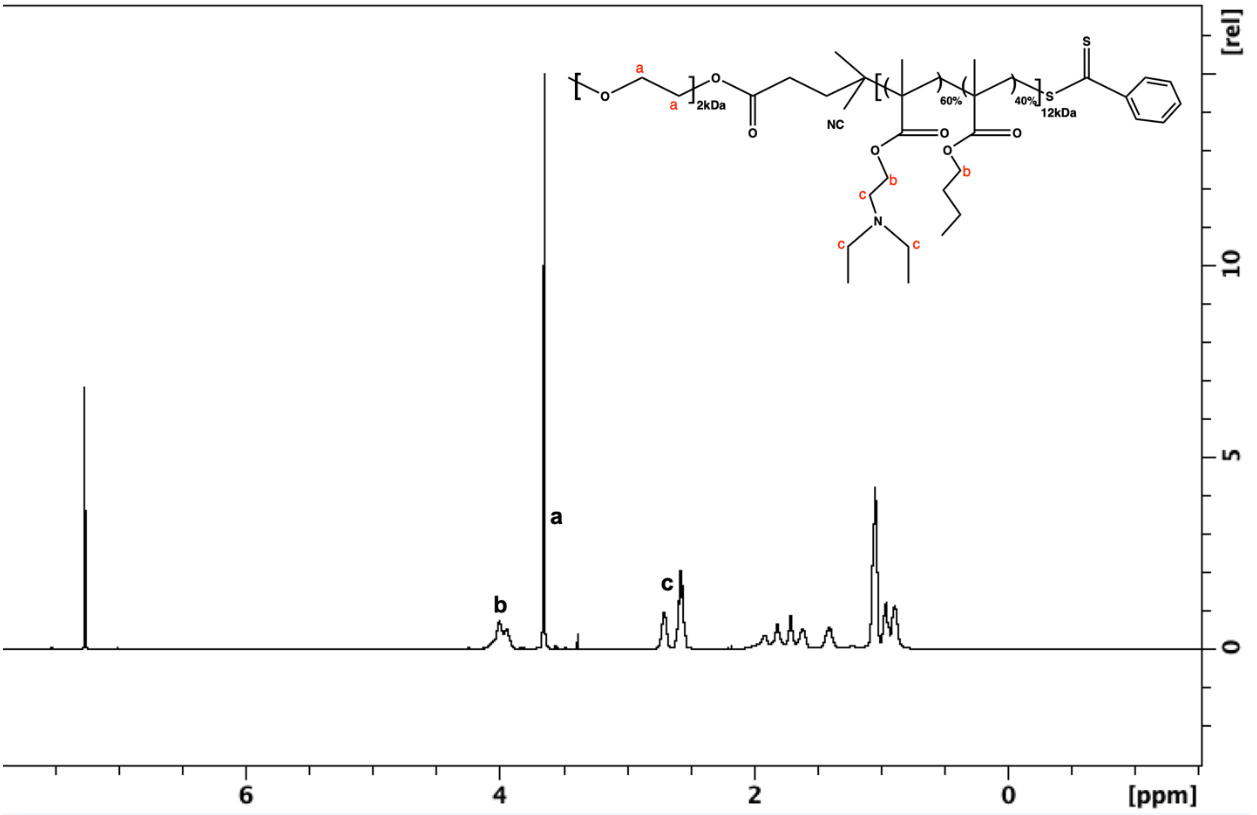


**Figure S11. Polymer Characterization.** Representative $^{1}$H-NMR (CDCl3) of (PEG)-*block*-(DEAEMA-*co*-BMA) diblock copolymer.

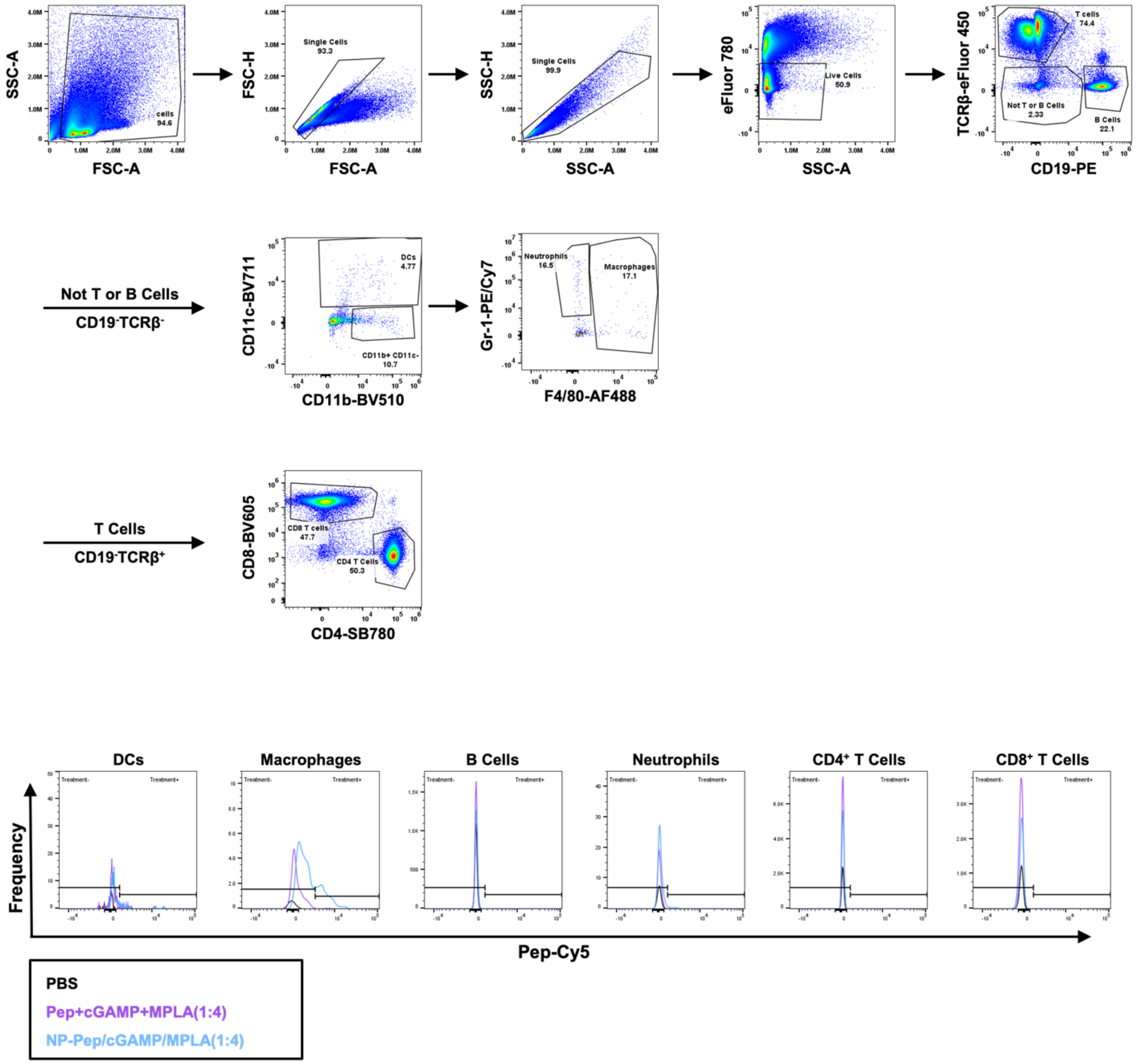


**Figure S12. Gating Strategy for Flow Cytometric Analysis of Cy5-OVA Uptake in Immune Cells in Lymph Node.** Events were first gated on lymphocytes (FSC-A vs SSC-A), then single cells (FSC-A vs FSC-H, then SSC-A vs SSC-H) and then live cells (eFluor negative cells). B cells were then gated as TCRβ$^-$CD19$^+$ cells. In this same plot T cells were gated out as TCRβ$^+$CD19$^-$ cells and the rest of the immune cells were gated out as TCRβ$^-$CD19$^-$ cells. Within the T cell (TCRβ$^+$CD19$^-$) gate, CD8$^+$ T cells were gated as CD8$^+$CD4$^-$ and CD4$^+$ T cells were gated as CD8$^-$CD4$^+$. Within the TCRβ$^-$CD19$^-$ gate, dendritic cells were gated on CD11c$^+$ cells, and neutrophils and macrophages were gated on CD11c$^-$CD11b$^+$ cells. Within this gate macrophages were gated as F4/80$^+$ and neutrophils were gated as F4/80$^-$GR-1$^+$ cells. Representative histograms of Cy5-OVAp within these immune cell populations is shown.

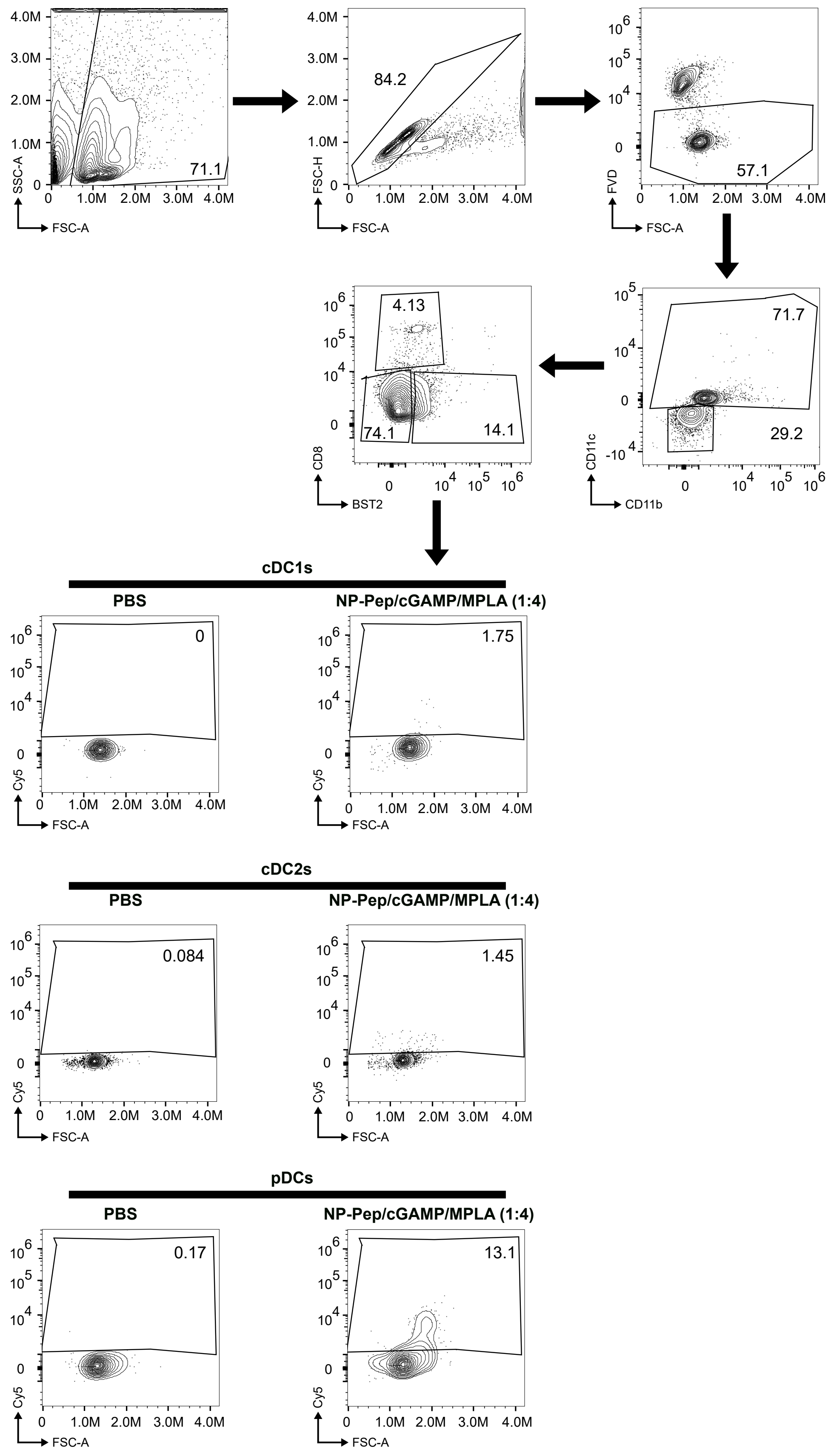
SSC-A
FSC-A
71.1
FSC-H
84.2
FVD
57.1
CD11c
CD11b
71.7
29.2
CD8
BST2
4.13
74.1
14.1
cDC1s
PBS
NP-Pep/cGAMP/MPLA (1:4)
Cy5
0
1.75
cDC2s
0.084
1.45
pDCs
0.17
13.1

**Figure S13. Gating Strategy for Flow Cytometric Analysis of Cy5-OVA Uptake in Dendritic Cell Subsets in Lymph Node.** Events were first gated on lymphocytes (FSC-A vs SSC-A), then single cells (FSC-A vs FSC-H) and then live cells (FVD negative cells). Dendritic cells were then gated on $CD11c^{+}$ cells. From the DCs, cDC1s were gated as $CD8^{+}$, cDC2s were gated as $CD8^{-}BST2^{-}$, and pDCs were gated as $CD8^{-}BST2^{+}$. The percentage of Cy5-OVAp was then gated within these DC subsets.

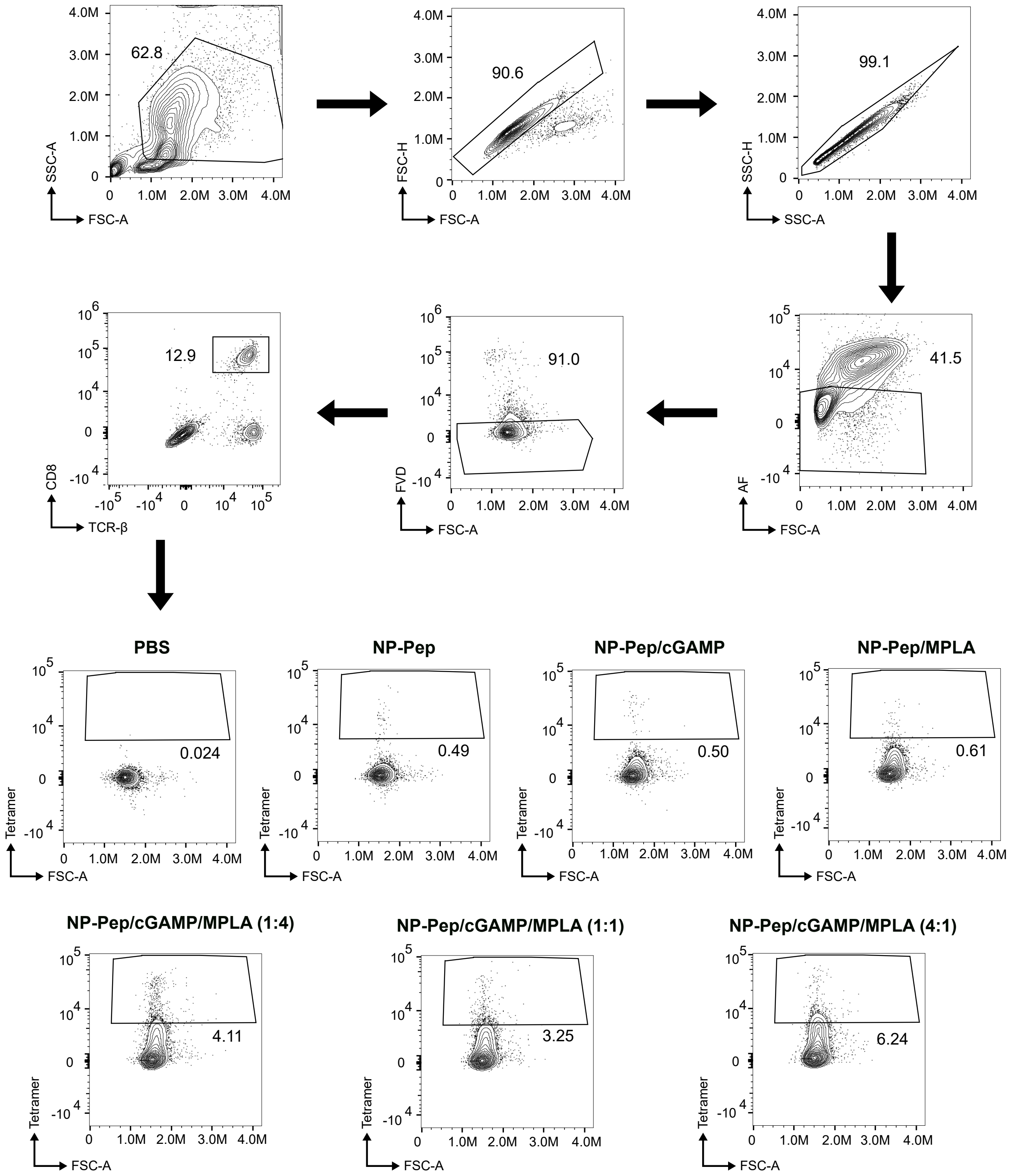


**Figure S14. Gating Strategy for Flow Cytometric Analysis Antigen-Specific T Cell Response in Spleen via Staining with PE-labeled pOVA/MHC-I Tetramer.** Events were first gated on lymphocytes (FSC-A vs SSC-A), then single cells (FSC-A vs FSC-H, then SSC-A vs SSC-H), then low autofluorescence cells, then live cells (FVD negative cells). Cells were gated on TCRβ$^+$CD8$^+$ T cells, and finally on tetramer$^+$ cells.

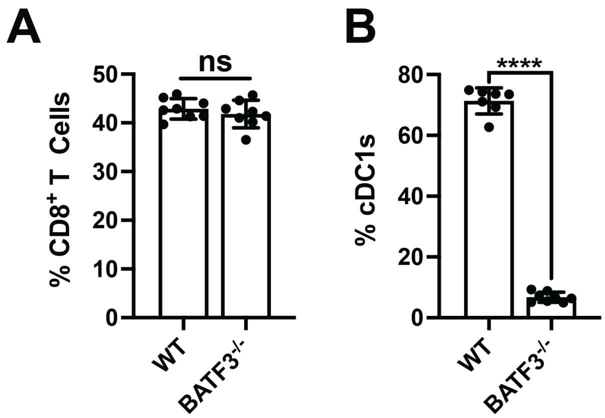


**Figure S15. Validation of Batf3-/- Model.** Flow cytometric evaluation of percentage of $CD8^+$ T cells (A) and cDC1 cells (B) in spleen, comparing C57/Bl6J wild type (WT) and $Batf3^{-/-}$ mouse strains to validate model (mean +/- SD; n=8; ****P<0.0001; unpaired t-test).

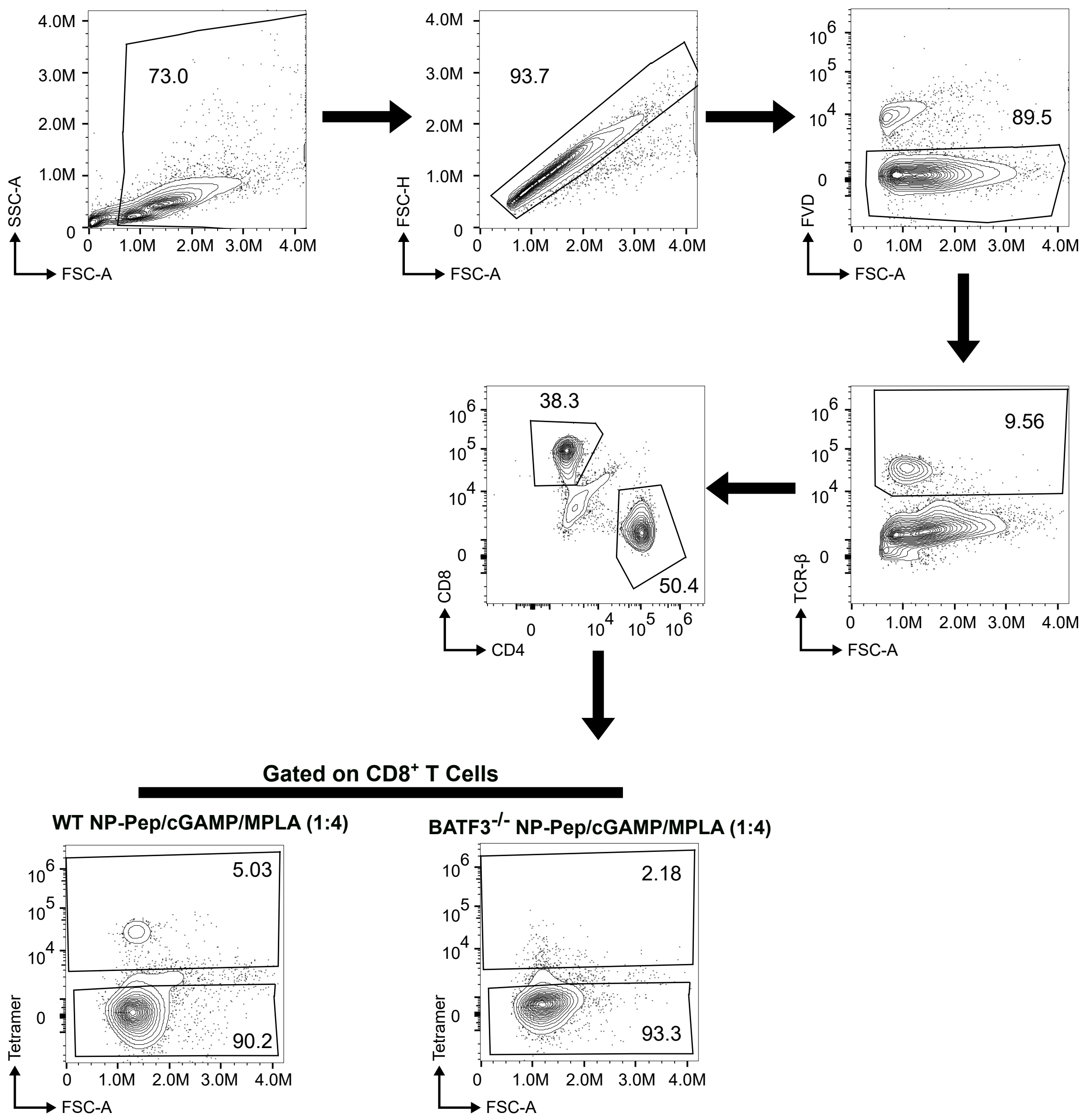


**Figure S16. Gating Strategy for Flow Cytometric Analysis Antigen-Specific T Cell Response in Spleen via Staining with PE-labeled pOVA/MHC-I Tetramer.** Events were first gated on lymphocytes (FSC-A vs SSC-A), then single cells (FSC-A vs FSC-H), then live cells (FVD negative cells). Cells were then gated on T cells (TCR-$\beta^{+}$), then CD8$^{+}$ T cells (CD8$^{+}$CD4$^{-}$), and finally on tetramer$^{+}$ cells.

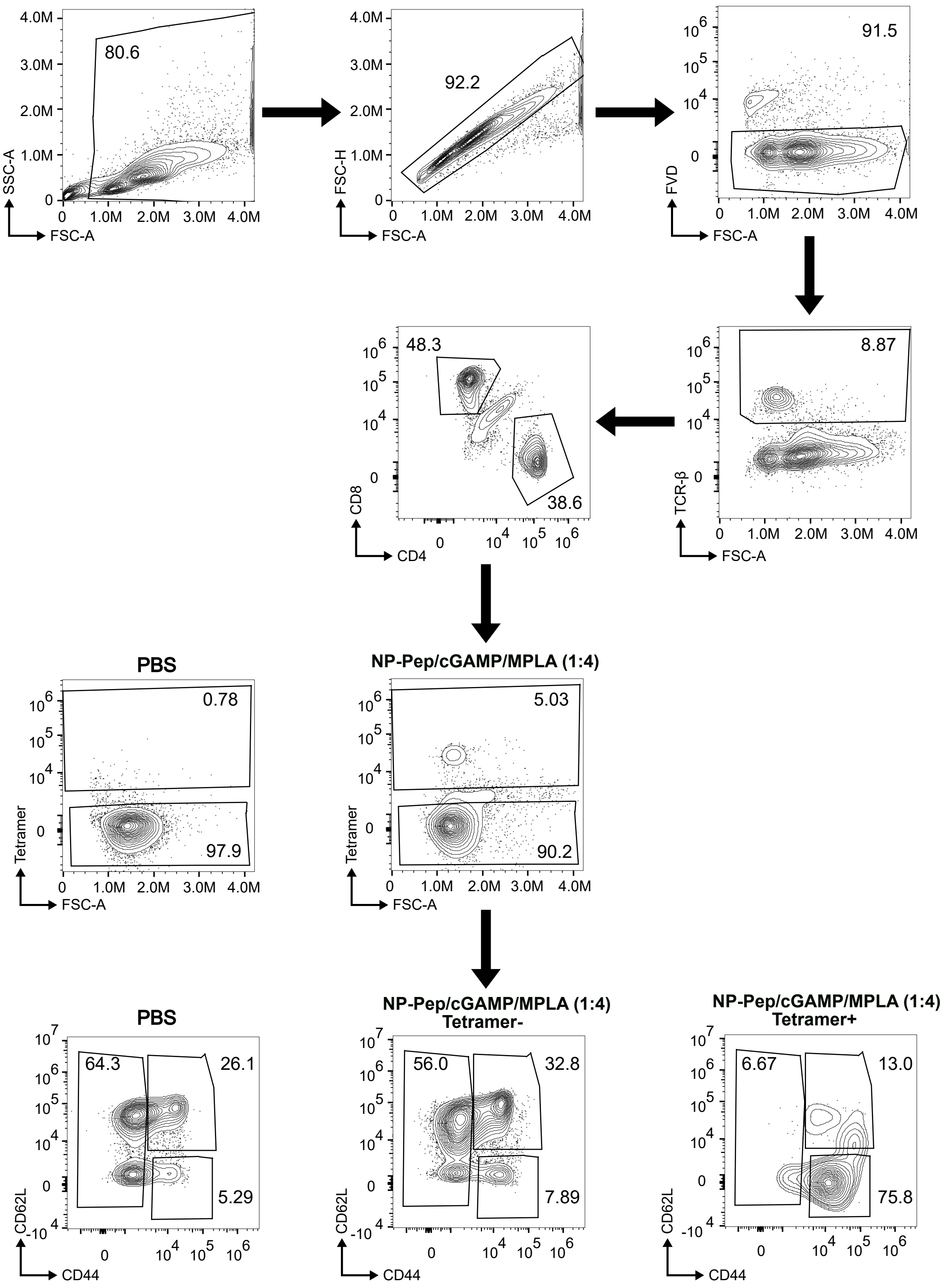


**Figure S17. Gating Strategy for Flow Cytometric Analysis of Memory Phenotype of CD8⁺ T Cells Response in Spleen.** Events were first gated on lymphocytes (FSC-A vs SSC-A), then single

cells (FSC-A vs FSC-H), then live cells (FVD negative cells). Cells were then gated on T cells (TCR-β$^+$), then CD8$^+$ T cells (CD8$^+$CD4$^-$), and then on tetramer$^-$ and tetramer$^+$ subsets. Tetramer$^-$ cells from PBS treated mice, and both tetramer$^-$ and tetramer$^+$ subsets of mice treated with vaccine formulation were then gated on CD62L vs CD44 to evaluate central memory (CD44$^+$CD62L$^+$) and effector memory (CD44$^+$CD62L$^-$) subsets.

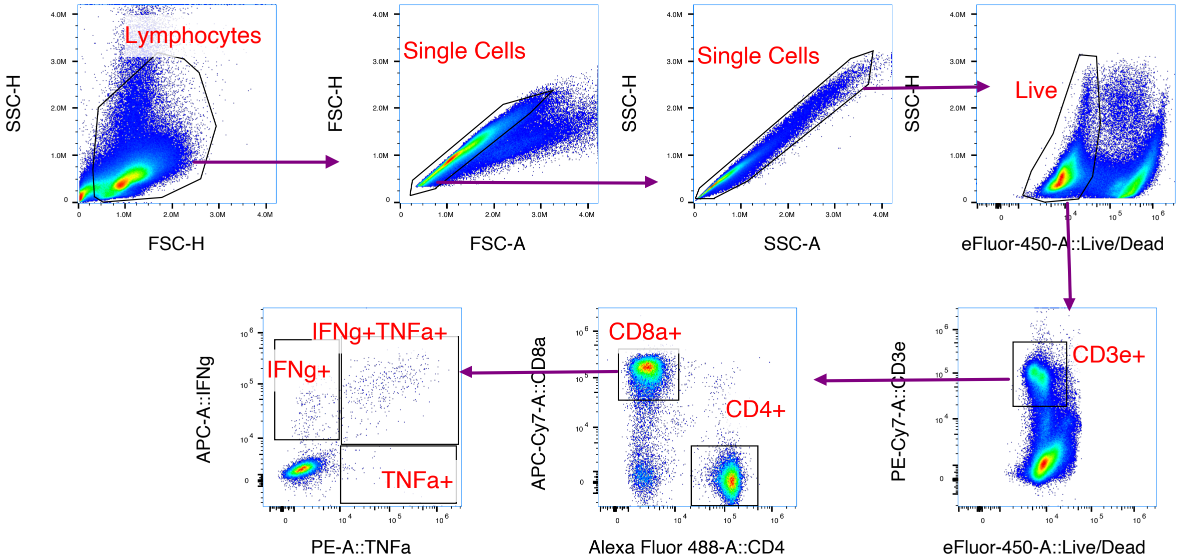


**Figure S18. Gating Strategy for Intracellular Cytokine Staining of CD8$^+$ T Cells in Spleen.** Events were first gated on lymphocytes (FSC-A vs SSC-A), then single cells (FSC-A vs FSC-H, then SSC-A vs SSC-H), then live cells (eFluor 450 negative cells). Cells were gated on T cells (CD3ε$^+$), then CD8$^+$ T cells (CD8$^+$CD4$^-$), and finally on IFNγ$^+$TNFα$^+$ cells.
